# Investigating the oral microbiome in retrospective and prospective cases of prostate, colon, and breast cancer

**DOI:** 10.1038/s41522-023-00391-7

**Published:** 2023-05-01

**Authors:** Jacob T. Nearing, Vanessa DeClercq, Morgan G. I. Langille

**Affiliations:** 1 Department of Microbiology and Immunology, Dalhousie University, Halifax, NS Canada; 2 Department of Pharmacology, Dalhousie University, Halifax, NS Canada

**Keywords:** Microbiome, Bacteria, Microbiology

## Abstract

The human microbiome has been proposed as a potentially useful biomarker for several cancers. To examine this, we made use of salivary samples from the Atlantic Partnership for Tomorrow’s Health (PATH) project and Alberta’s Tomorrow Project (ATP). Sample selection was divided into both a retrospective and prospective case control design examining prostate, breast, and colon cancer. In total 89 retrospective and 260 prospective cancer cases were matched to non-cancer controls and saliva samples were sequenced using 16S rRNA gene sequencing. We found no significant differences in alpha diversity. All beta diversity measures were insignificant except for unweighted UniFrac profiles in retrospective breast cancer cases and weighted UniFrac, Bray-Curtis and Robust Atchinson’s distances in colon cancer after testing with age and sex adjusted MiRKAT models. Differential abundance (DA) analysis showed several taxa that were associated with previous cancer in all three groupings. Only one genus (Clostridia *UCG-014*) in breast cancer and one ASV (*Fusobacterium periodonticum*) in colon cancer was identified by more than one DA tool. In prospective cases three ASVs were associated with colon cancer, one ASV with breast cancer, and one ASV with prostate cancer. Random Forest classification showed low levels of signal in both study designs in breast and prostate cancer. Contrastingly, colon cancer did show signal in our retrospective analysis (AUC: 0.737) and in one of two prospective cohorts (AUC: 0.717). Our results indicate that it is unlikely that reliable microbial oral biomarkers for breast and prostate cancer exist.. However, further research into the oral microbiome and colon cancer could be fruitful.

## Introduction

The oral microbiome is a highly diverse microbial community that is shaped by several different dietary, anthropometric and lifestyle choices^1,2^. Recent works have shown that this community of microbes plays important roles in both oral and systemic health, suggesting that it may harbor potential biomarkers of disease^3^. However, research on the relation of the oral microbiome and some of the most common cancers such as prostate, colon, and breast cancer are limited due to their focus on the gut microbiota or organ of interest. However, we believe that saliva could be an ideal sample type for microbial biomarker detection in these cancers due to its ease of collection, storage, and transportation. Moreover, previous works suggest that the oral microbiome may be associated with various cancers such an pancreatic and colon^4,5^.

Of these cancers, prostate cancer has arguably received the least amount of attention within the microbiome field. Indeed, studies on the microbial communities of healthy prostate tissue have given conflicting results, with some indicating the presence of bacteria and others finding no evidence of bacterial inhabitants^6^. However, in the context of prostate cancer, multiple works have demonstrated evidence for bacterial communities in tumors and benign tissue^7–9^. Despite these findings the association between disease and specific bacteria within prostate tissue remains inconsistent. Although, ultimately these studies have point toward similar communities being detected in tumors and benign tissue^7–9^.

Several smaller studies have examined the relationship between the gut microbiome and prostate cancer with mixed results. Two studies by Liss et al., and Golombos et al., found higher levels of Bacteroidetes in individuals with prostate cancer indicating a potential linkage between the disease and this broad taxonomic group^10,11^. Furthermore, work by Matsushita et al., in Japanese men with high-Gleason prostate cancer linked an enrichment of short chain fatty acid producing bacteria in the gut and cancer status^12^. However, other works by Alanee et al. and Katz et al., have found no significant differences between individuals with and without prostate cancer^13,14^ leading to mixed results on the exact relation between prostate cancer and gut microbiome composition.

To the best of our knowledge only one study has examined the potential linkage between the oral microbiome and prostate cancer, despite previous works linking periodontitis with the likelihood of prostate cancer development^15,16^. Indeed work by Estemalik et al., showed evidence of the same bacterial species being found within both the oral cavity and prostatic secretions of 70% of patients (*n* = 24) with chronic prostatitis or benign prostatic hyperplasia^17^. Highlighting the potential for associations between the oral microbiome and prostate cancer.

Studies on breast cancer and the human microbiome have shown variable results with some studies suggesting associations between disease status and microbial community composition within the gut, breast tissue, and urine^18^. Indeed, early work on the gut microbiota and breast cancer suggested the presence of an “estrobolome”^19,20^ which considers the potential for microbial community members to process estrogen related metabolites. From these works it has been suggested that the estrobolome may play a role in the risk of breast cancer development through the control of recirculating estrogen levels^19,21,22^.

Investigation into this hypothesis through examining the relationship between the gut microbiome and breast cancer status has shown varying results. For example, work by Goedert et al., found reduced microbial diversity within the gut of post-menopausal breast cancer patients, however, work in 2018 by Zhu et al., reported significant findings in the opposite direction^23,24^. Similarly, several gut microbes have been associated with breast cancer status depending on study and menopausal status^23–27^. However, due to the high variability of results between studies and the lack of non-sequenced based validation, no strong conclusions have been made on the role of any specific gut taxon and breast cancer risk.

In addition to the gut, several studies have examined breast cancer status and microbial communities within and on breast tissue and urine. Although, these studies just like the gut, have also shown variable results indicating the need for further work within the field^18^. Interestingly, in the case of the oral microbiome, despite work showing that breast cancer is associated with periodontal disease^28^ to the best of our knowledge only two published studies have examined its relation with cancer status. Early work by Wang et al., examining oral rinses from individuals in the United States found no differences in community composition or specific taxa^29^. However, recent work by Wu et al., did show differences in both microbial diversity and specific taxon within salivary samples from the Ghana Breast Health Study^30^. These differing results highlight the need for further investigation into the oral microbiome of breast cancer patients.

Finally, one of the most well studied cancers in the context of the human microbiome is colon cancer. Numerous studies have been conducted on the relationship between colon cancer and the gut microbiome showing significant differences in community composition^31^. Similarly work on the oral microbiome and colon cancer has also shown that shifts in community composition are associated with disease (Komiya et al.,^32^; Y. Wang et al.,^33^; Y. Yang et al.,^34^, Flemer et al.,^4^). For example, Flemer et al., found that oral microbiome composition could classify individuals with or without colon cancer with an area under the receiver operator curve (AUROC) of 0.91^4^. Furthermore, their work along with others has also shown that many taxa commonly attributed to the oral cavity are enriched in the gut microbiome of colon cancer patients^4,35^.

However, despite the work presented above there still remains large knowledge gaps in our understanding of the oral microbiome’s applicability to population screening for prostate, breast, and colon cancer. For this reason, we were interested in investigating these three cancers at a population level in both a case-control retrospective and prospective study design. To do this we leveraged two different population cohorts the Atlantic Partnership for Tomorrow’s Health (PATH), and Alberta’s Tomorrow Project (ATP). From these two cohorts we selected saliva samples from both retrospective cases and prospective cases of prostate, breast, and colon cancer. This unique study design allowed us to investigate the relationship of these cancers with the oral microbiome both before and after diagnosis allowing us to investigate whether associated exist both before and after diagnosis.

## Results

### Investigation of the oral microbiome in retrospective cases of breast, prostate, and colon cancer

**Fig. 1:**
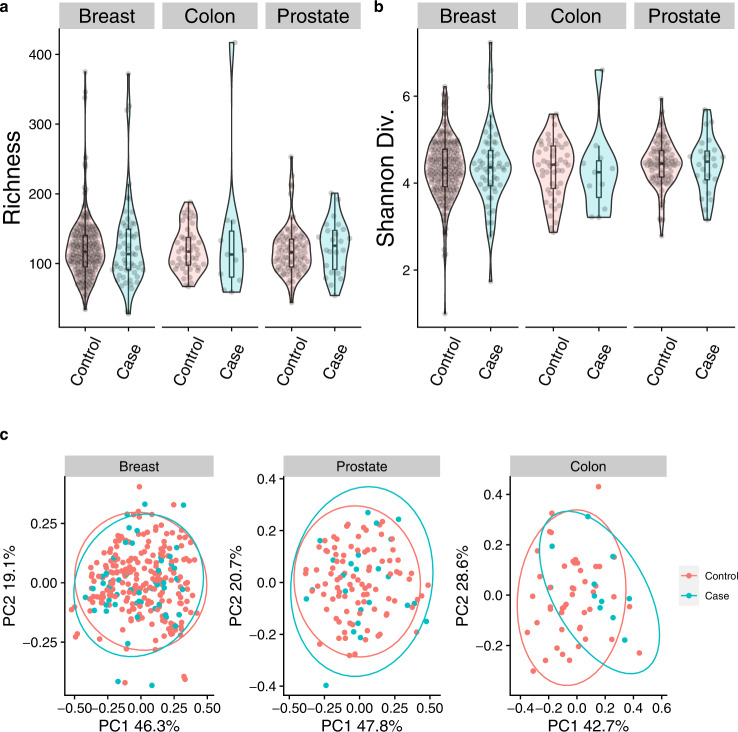
Oral microbiome diversity metrics of retrospective cases of breast, colon, and prostate cancer in the retrospective Atlantic PATH cohort. Comparing microbial diversity of non-cancer matched controls to case samples of retrospective prostate, colon and breast cancer showed no significant differences in alpha diversity as measured by richness (**a**), and Shannon diversity (**b**). Principal coordinates of analysis comparing weighted UniFrac profiles between cancer cases and non-cancer controls. Colon Cancer was found to be significant in an age and sex adjusted MiRKAT test (*p* = 0.027), however remained insignificant in PERMANOVA testing of the same co-variates (*p* = 0.122) (**c**). The interquartile range (IQR) of boxplots represent the 25th and 75th percentiles while maxima and minima represent the maximum and minimum values outside 1.5 times the IQR. The central line represents the median within that group.

First, oral microbiome diversity trends between case and control samples were examined within retrospective cases of breast, prostate, and colon cancer within the Atlantic PATH cohort (Table 1). In total we examined four different alpha diversity metrics: richness, Shannon diversity, Evenness, and Faith’s Phylogenetic Diversity while controlling for DNA extraction batch. Investigation into these four metrics did not show any significant differences in alpha diversity between case and non-cancer controls in breast, prostate, or colon cancer (*p* > 0.05) (Fig. 1, Supplementary Fig. 1, Table 2). Further adjustment of all four alpha diversity models with either partial adjusted (age and sex), or full adjusted (sex, height, waist-hip ratio, and daily vegetable servings) further showed no significant associations (*p* > 0.05) (Table 2). Subsequently, we also compared four different beta diversity metrics, three that consider weighted abundances; weighted UniFrac, Bray-Curtis dissimilarity, robust Aitchison’s distance, and one that considers presence/absence; unweighted UniFrac. When comparing cases of each cancer type to matched non-cancer controls, we found no significant differences (*p* > 0.05) in any weighted beta diversity metrics using unadjusted PERMANOVA analysis or a microbiome regression-based kernel association test (MiRKAT) (*p* > 0.05) (Fig. 1, Supplementary Fig. 2, Table 3). However, after partially adjusting MiRKAT models for age and sex we did find significant differences in weighted UniFrac (*p* = 0.027), Bray-Curtis dissimilarity (*p* = 0.007) and Robust Atchinson’s distance (0.028) between colon cancer cases and non-cancer matched controls (Table 2). Moreover, when comparing unweighted Unifrac distances we did find a significant difference between breast cancer cases and controls in unadjusted (PERMANOVA: *r*^2^ = 0.007, *p* = 0.011) (MiRKAT: *p* = 0.014) as well as partially adjusted and fully adjusted models. (Supplementary Fig. 2A, Table 3). This breast cancer association remained significant for PERMANOVA models after partial (*p* = 0.018) and full adjustment (*p* = 0.014) but went above our nominal *p* value when tested with MiRKAT models (partial *p* = 0.119; full *p* = 0.099).

**Table 1:** Cohort characteristics for investigation of the oral microbiome in cases of breast, colon, and prostate cancer.

Cancer type	Breast cancer		Prostate cancer		Colon cancer	
Case vs. Control	Case	Control	Case	Control	Case	Control
Atlantic PATH cohort—Retrospective cases						
Number of samples	54	218	24	92	11	47
Sex (% female)	100%	100%	0%	0%	53%	53%
Mean Age-years (SD)	57.5 (8.15)	55.6 (8.16)	^a^ 60.6 (5.92)	57.7 (6.36)	59.5 (10.2)	56.9 (9.21)
Mean BMI-kg/m^2^ (SD)	29.0 (5.01)	28.3 (4.72)	29.6 (3.27)	28.8 (3.49)	28.9 (4.16)	28.5 (4.13)
Standing Height-cm (SD)	^a^ 161 (5.25)	163 (6.46)	174 (6.21)	177 (7.58)	170 (10.4)	170 (8.53)
Waist-Hip Ratio (SD)	0.871 (0.114) 2 Missing	0.848 (0.066)	0.957 (0.053) 1 Missing	0.953 (0.061)	0.930 (0.097)	0.903 (0.064)
Daily Vegetable Servings (SD)	2.75 (1.14) 2 Missing	2.79 (1.60) 1 Missing	2.42 (1.61)	2.09 (1.27)	2.64 (1.63)	2.19 (1.24)
% Current Smoker	0	0	0	0	0	0
Median Time Since Diagnosis (years)	6	N/A	4	N/A	5	N/A
Atlantic PATH cohort—Prospective cases						
Number of samples	54	54	28	28	10	10
Sex (% female)	100%	100%	0%	0%	70%	70%
Mean Age-years (SD)	56.6 (7.74)	56.9 (8.38)	60.6 (4.43)	61.0 (4.93)	60.4 (7.88)	60.7 (8.25)
Mean BMI-kg/m^2^ (SD)	26.9 (5.25)	26.9 (5.45)	28.3 (4.46)	28.1 (4.72)	27.9 (6.08)	28.3 (5.80)
Standing Height-cm (SD)	164 (4.88)	162 (5.99)	178 (7.98)	175 (6.55)	164 (5.47)	169 (12.6)
Waist-Hip Ratio (SD)	0.839 (0.072)	0.856 (0.066)	0.970 (0.060)	0.975 (0.076)	0.780 (0.284)	0.876 (0.089)
Daily Vegetable Servings (SD)	2.42 (2.18) 6 Missing	3.02 (1.45) 3 Missing	2.12 (0.971) 3 Missing	2.22 (1.62) 5 Missing	3.11 (1.27) 1 Missing	2.38 (1.51) 2 Missing
% Current Smoker	1.85%	3.70%	0%	3.57%	0%	0%
Median Time Before Diagnosis (years)	4	N/A	3.5	N/A	3	N/A
ATP cohort—Prospective cases						
Number of samples	82	82	64	64	22	22
Sex (% female)	100%	100%	0%	0%	50%	50%
Mean Age-years (SD)	57.7 (8.74)	57.7 (8.70)	63.2 (6.92)	63.2 (6.88)	60.0 (10.0)	60.0 (9.99)
Mean BMI-kg/m^2^ (SD)	28.3 (6.00) 11 Missing	26.9 (5.60) 10 Missing	27.3 (4.3) 7 Missing	28.2 (4.94) 5 Missing	30.6 (6.94) 1 Missing	27.1 (4.49) 3 Missing
Standing Height-cm (SD)	162 (6.88) 11 Missing	163 (5.57) 9 Missing	177 (6.74) 7 Missing	178 (6.96) 5 Missing	167 (8.32) 1 Missing	167 (7.58) 3 Missing
Waist-Hip Ratio (SD)	0.912 (0.0862) 13 Missing	0.901 (0.047) 11 Missing	0.967 (0.060) 9 Missing	0.956 (0.051) 8 Missing	0.958 (0.063) 2 Missing	0.939 (0.044) 5 Missing
Daily Vegetable Servings (SD)	^a^ 2.85 (1.66) 1 Missing	3.55 (1.80) 4 Missing	2.02 (1.15) 2 Missing	2.18 (1.60) 2 Missing	2.76 (1.67) 1 Missing	2.86 (1.59) 1 Missing
% Current Smoker	1.22%	1.22%	15.6%	15.6%	9.09%	9.09%
Median Time Before Diagnosis-years	4.28	N/A	3.10	N/A	2.98	N/A

^a^Indicates significant difference from control within the same cancer type (*p* < 0.05). *SD* Standard deviation.

**Table 2:** *P* value results from unadjusted, partially adjusted, and fully adjusted linear models comparing alpha diversity between case and non-cancer matched controls across each dataset.

Cancer type:		Breast cancer	
Model	Unadjusted	Partial adjusted	Fully adjusted
Statistic:	*p* value	*p* value	*p* value
Atlantic PATH cohort—Retrospective cases			
Faith’s PD	0.224	0.206	0.179
Richness	0.996	0.958	0.857
Shannon	0.256	0.24	0.272
Evenness	0.343	0.292	0.297
Atlantic PATH cohort—Prospective cases			
Faith’s PD	0.258	0.266	0.72
Richness	0.482	0.495	0.963
Shannon	0.933	0.902	0.459
Evenness	0.668	0.634	0.32
ATP cohort—Prospective cases			
Faith’s PD	0.308	0.303	0.525
Richness	0.382	0.369	0.719
Shannon	0.603	0.591	0.561
Evenness	0.151	0.147	0.221
Cancer Type:		Prostate Cancer	
Atlantic PATH cohort—Retrospective cases			
Faith’s PD	0.486	0.555	0.761
Richness	0.816	0.963	0.831
Shannon	0.788	0.892	0.682
Evenness	0.799	0.826	0.724
Atlantic PATH cohort—Prospective cases			
Faith’s PD	0.679	0.664	0.642
Richness	0.584	0.56	0.489
Shannon	0.905	0.841	0.913
Evenness	0.872	0.935	0.792
ATP cohort—Prospective cases			
Faith’s PD	0.21	0.204	0.115
Richness	0.152	0.147	0.084
Shannon	0.312	0.305	0.136
Evenness	0.48	0.476	0.273
Cancer Type:		Colon Cancer	
Atlantic PATH cohort—Retrospective cases			
Faith’s PD	0.249	0.33	0.254
Richness	0.722	0.751	0.671
Shannon	0.647	0.627	0.575
Evenness	0.604	0.627	0.544
Atlantic PATH cohort—Prospective cases			
Faith’s PD	0.646	0.618	0.216
Richness	0.375	0.376	0.2909
Shannon	0.685	0.661	0.9
Evenness	0.967	0.95	0.49
ATP cohort—Prospective cases			
Faith’s PD	0.528	0.527	0.908
Richness	0.725	0.728	0.96
Shannon	0.365	0.338	0.705
Evenness	0.34	0.37	0.685

**Table 3:** *P* value results from unadjusted, partially adjusted, and fully adjusted PERMANOVA and MiRKAT analysis comparing beta diversity between case and non-cancer matched controls across each dataset.

Cancer type:			Breast cancer			
Model	Unadjusted		Partial adjusted		Fully adjusted	
Statistic:	PERMANOVA *p* value	MiRKAT *p* value	PERMANOVA *p* value	MiRKAT *p* value	PERMANOVA *p* value	MiRKAT *p* value
Atlantic PATH cohort—Retrospective cases						
Weighted UniFrac	0.483	0.463	0.544	0.678	0.545	0.657
Unweighted UniFrac	^a^ 0.011	^a^ 0.014	^a^ 0.018	0.119	^a^ 0.014	0.099
Bray-Curtis	0.325	0.373	0.372	0.441	0.49	0.483
Robust Atchinson’s	0.408	0.369	0.402	0.35	0.456	0.243
Atlantic PATH cohort—Prospective cases						
Weighted UniFrac	0.188	0.208	0.238	0.214	0.255	0.191
Unweighted UniFrac	0.334	0.371	0.338	0.374	0.725	0.809
Bray-Curtis	0.603	0.629	0.654	0.641	0.648	0.652
Robust Atchinson’s	0.654	0.646	0.65	0.657	0.817	0.778
ATP cohort—Prospective cases						
Weighted UniFrac	0.912	0.922	0.911	0.921	0.478	0.519
Unweighted UniFrac	0.487	0.46	0.457	0.452	0.366	0.39
Bray-Curtis	0.194	0.206	0.184	0.201	0.338	0.377
Robust Atchinson’s	0.201	0.22	0.205	0.214	0.179	0.175
Cancer Type:		Prostate Cancer				
Atlantic PATH cohort—Retrospective cases						
Weighted UniFrac	0.69	0.604	0.757	0.863	0.911	0.916
Unweighted UniFrac	0.337	0.383	0.423	0.1	0.509	0.053
Bray-Curtis	0.47	0.45	0.475	0.78	0.457	0.825
Robust Atchinson’s	0.164	0.179	0.246	0.689	0.172	0.865
Atlantic PATH cohort—Prospective cases						
Weighted UniFrac	0.966	0.981	0.967	0.985	0.758	0.644
Unweighted UniFrac	0.835	0.818	0.85	0.829	0.881	0.857
Bray-Curtis	0.723	0.734	0.71	0.739	0.516	0.472
Robust Atchinson’s	0.596	0.592	0.552	0.584	0.284	0.258
ATP cohort—Prospective cases						
Weighted UniFrac	0.09	0.085	0.073	0.084	0.08	0.08
Unweighted UniFrac	0.711	0.66	0.727	0.658	0.338	0.32
Bray-Curtis	0.442	0.473	0.49	0.47	0.337	0.333
Robust Atchinson’s	0.254	0.252	0.251	0.254	0.106	0.123
Cancer Type:			Colon Cancer			
Atlantic PATH cohort—Retrospective cases						
Weighted UniFrac	0.139	0.283	0.122	^a^ 0.027	0.577	0.106
Unweighted UniFrac	0.222	0.448	0.209	0.558	0.191	0.318
Bray-Curtis	0.116	0.367	0.165	^a^ 0.007	0.293	0.056
Robust Atchinson’s	0.336	0.452	0.387	^a^ 0.028	0.348	0.127
Atlantic PATH cohort—Prospective cases						
Weighted UniFrac	0.722	0.768	0.745	0.769	0.536	0.996
Unweighted UniFrac	0.692	0.663	0.668	0.663	0.066	0.164
Bray-Curtis	0.49	0.521	0.541	0.507	0.433	0.794
Robust Atchinson’s	0.603	0.631	0.637	0.641	0.606	0.816
ATP cohort—Prospective cases						
Weighted UniFrac	0.531	0.494	0.507	0.48	0.987	0.973
Unweighted UniFrac	0.432	0.408	0.421	0.43	0.797	0.799
Bray-Curtis	0.7	0.706	0.735	0.686	0.97	0.92
Robust Atchinson’s	0.152	0.156	0.193	0.19	0.542	0.458

^a^Indicates significant difference from control within the same cancer type (*p* < 0.05).

To investigate whether we were missing an effect of cancer status due to the passage of time since diagnosis, we correlated each alpha diversity metric to this variable (Supplementary Fig. 3). We found no significant relationships except for colon cancer which showed a positive association between time passed since diagnosis and alpha diversity (rho = 0.62, *p* = 0.04). We also re-examined samples that were within 6 years of diagnosis to see whether more significant microbiome effects were present closer to cancer diagnosis. Examining these samples showed only minor differences to our original analysis. In this time filter analysis, we found a significant decrease in richness in colon cancer samples compared to matched controls (*p* = 0.012) that remained significant after partial or full model adjustment (*p* = 0.024, *p* = 0.033) (Supplementary Fig. 4). We additionally found a significant association between breast cancer cases and non-cancer matched controls in robust Aitchison’s distance (*p* = 0.024) which remained significant in partially adjusted models (age and sex) (*p* = 0.024).

**Fig. 2:**
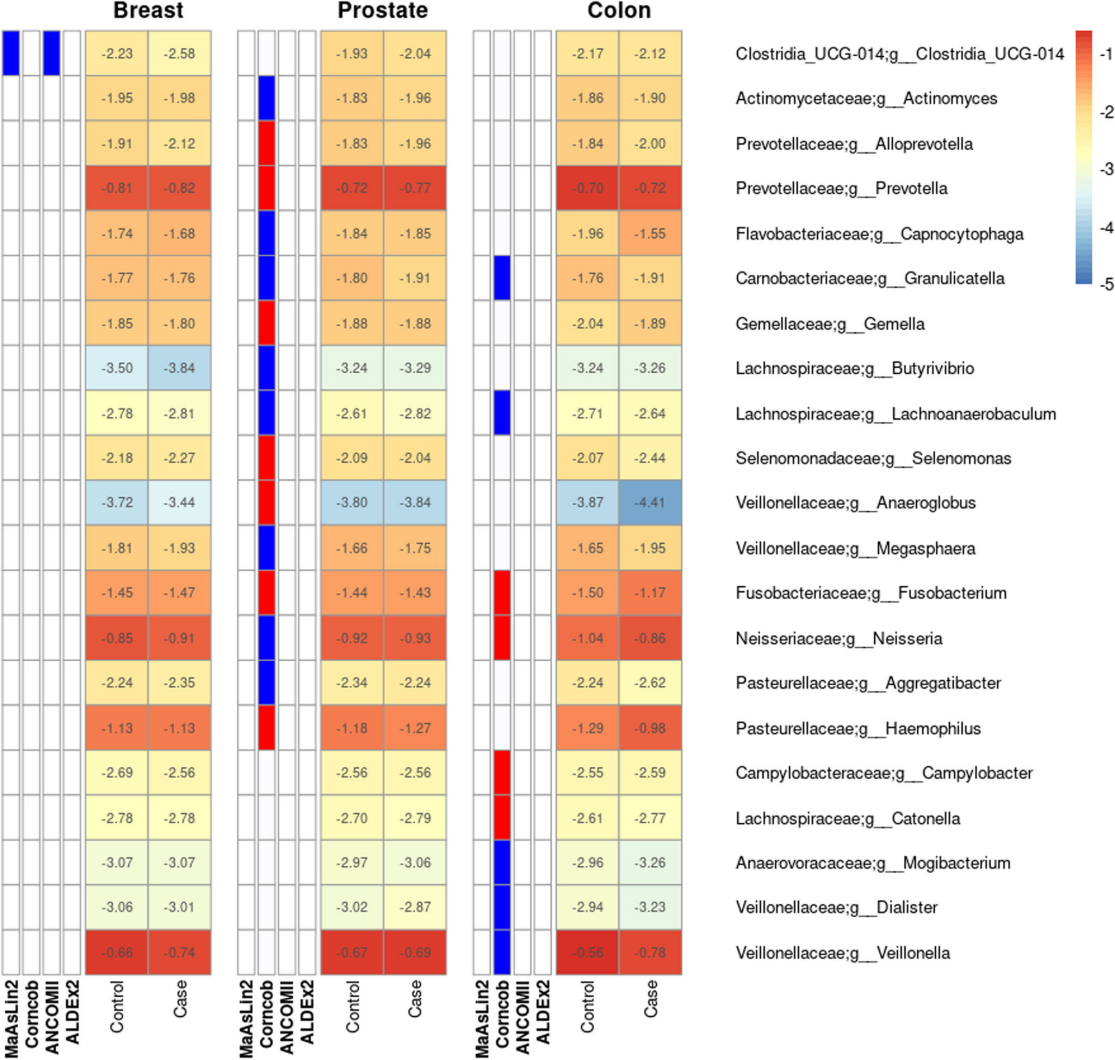
Several genera are detected as differentially abundant in the oral microbiome of retrospective cases of prostate, colon and breast cancer in the Atlantic PATH cohort. The heatmap is divided by cancer type where the first four columns represent the detection of significant associations by one of four tools: MaAsLin2, Corncob, ANCOM-II, and ALDEx2. Blue bars in the first four columns of each subgroup represent a detected increase in control samples while red bars represent a detected increase in case samples. The final two columns within each cancer sub grouping represent the log10 mean relative abundance of each genus with red representing higher abundance values and blue representing lower abundance values. Overall, corncob found the largest number of associated genera with a single genus in breast cancer also being detected by ANCOM-II.

After comprehensively examining samples for differences in diversity we decided to conduct DA analysis to identify genera or ASVs that might be associated with having previously been diagnosed with cancer. Across all cancers examined we found a total of 21 genera and 29 ASV’s associated with one or more cancer diagnoses (Fig. 2, Supplementary Fig. 5). In breast cancer we found one genus Clostridia UCG-014 that was detected as being significantly lower in relative abundance in breast cancer samples by two separate DA tools (Fig. 2). We also identified an additional 3 ASVs one of which belonged within the Clostridia UCG-014 genera (Supplementary Fig. 5). Interestingly, the top BLAST hit within the Human Oral Microbiome database to the ASV classified within Clostridia UCG-014 only shared 88% sequence identity with its top alignment. Although this taxon has shown to be a dominant member within other areas of the human body such as gut^36^. The other two low abundance ASVs only detected by ANCOM-II were classified within Capnocytophaga and Bergeyella (Supplementary Fig. 5). Although only the ASV classified within Capnocytophaga showed >90% nucleotide identity to any taxa within the Human Oral Microbiome database with a top hit to the species *Capnocytophaga ochracea*.

In prostate cancer, corncob identified 15 genera and 24 ASVs that are potentially differentially abundant between case and control samples (Fig. 2, Supplementary Fig. 5). However, no other tools detected these taxa, and only six ASVs and one genus showed an effect size larger than a 1.5-fold change in mean relative abundance between case and control samples. Furthermore, in these low effect size associations, we found inconsistencies between corncob’s coefficient directionalities and the observed differences between mean relative abundances between case and control samples (Fig. 2, Supplementary Fig. 5).

In colon cancer we identified 9 genera and 9 ASVs as being differentially abundant between retrospective case and non-cancer matched control samples (Fig. 2, Supplementary Fig. 5). All these features were identified by corncob and a single ASV classified as Fusobacterium was additionally detected by ANCOM-II to be increased in colon cancer cases (Supplementary Fig. 5, Supplementary Fig. 6). Inspection into the identity of this ASV at lower taxonomic levels using sortmeRNA^37^ identified this ASV as potentially coming from the species *Fusobacterium periodonticum*. Further examination of differential taxa associated with all three cancers using partially or fully adjusted models showed similar results (Supplementary Figs. 7–8).

**Fig. 3:**
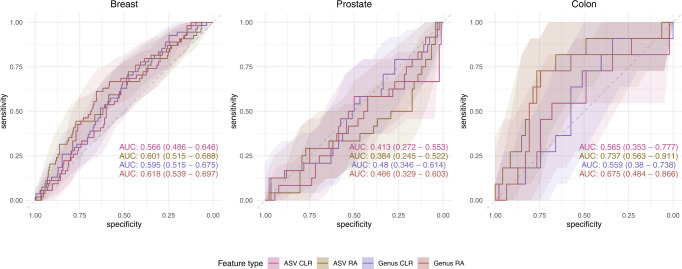
Random Forest classification of retrospective cases of breast, prostate, and colon cancer based on microbial taxonomic composition in the Atlantic PATH cohort. Receiver operator curves (ROC) showing the specificity and sensitivity of the classification of non-cancer matched controls and retrospective cases of breast, prostate or colon cancer. Models were constructed using 100-repeat 5-fold cross validation and hold-out performance was determined through taking the mean number of votes for each hold-out sample across all 100 repeats. Within each plot four different ROCs are represented showing the classification accuracy using ASVs or genera normalized with either total-sum-scaling or center-log-ratio abundance. Shaded areas represent 95% confidence intervals determined through 2000 bootstrap samplings.

Despite the relatively small taxonomic differences we were still interested in determining whether Random Forest classification models could pick up differences between case and control samples. Examining model performance on repeated hold-out sets during cross validation showed that both breast cancer and colon cancer models performed best. Contrastingly, all prostate cancer models regardless of input data type performed below an AUC of 0.5 (Fig. 3). Although breast cancer models were only modestly better with AUCs ranging from 0.566 to 0.618 (Fig. 3).

Colon cancer models performed the best although large confidence intervals were observed due to low sample size. Furthermore, colon cancer models showed highly variable performance depending on feature normalization. Models built using center-log-ratio normalizations performed only slightly better than random expectation with ASVs having an AUC of 0.565 (0.353–0.777 95% CI), and genera having an AUC of 0.559 (0.380–0.738 95% CI). However, models built using relative abundances showed stronger results with models having AUCs ranging from 0.675 to 0.737 (Fig. 3). Examining the top 10 most important features of each of these models showed relatively small decreases in accuracy for any single ASV/genera (Supplementary Figs. 9–10). Indeed, further inspection also showed that of these ASV/genera only 1 ASV overlapped with the previously identified differentially abundant taxon (Supplementary Fig. 5, Supplementary Fig. 9). This ASV was classified into the genera Veillonella and upon further inspection, best aligned to *Veillonella atypica* (100% identity) within the SILVA V138 database.

### Investigation of the oral microbiome in prospective cases of breast, prostate, and colon cancer

**Fig. 4:**
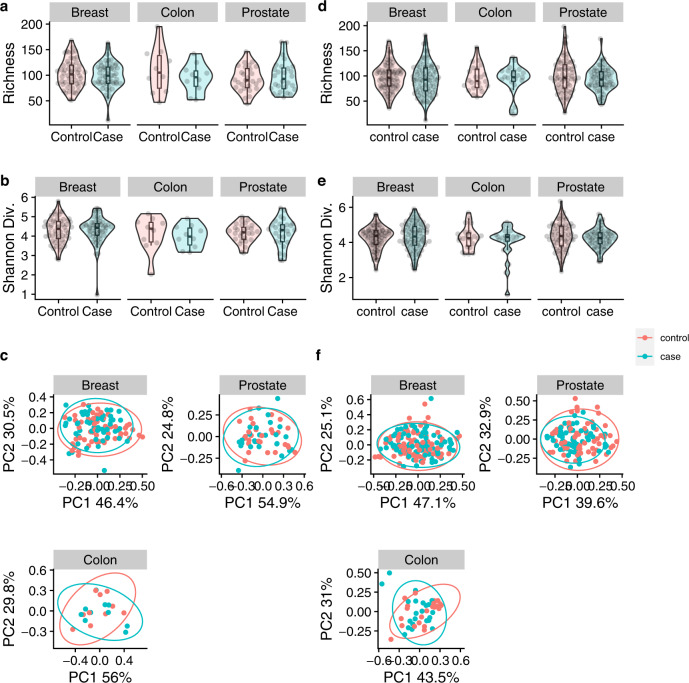
Oral microbiome diversity metrics of prospective cases of breast, prostate, and colon cancer in Atlantic PATH and ATP cohorts. Oral microbiome diversity analysis comparing non-cancer matched controls to prospective cases of colon breast and prostate cancer. Alpha diversity analysis as measured by richness (**a**) and Shannon diversity (**b**) as well as beta diversity measured as weighted UniFrac (**c**) showed no significant differences in the prospective PATH cohort. Similarly, no significant differences in richness (**d**), Shannon diversity (**e**) or weighted UniFrac (**f**) in any cancer type in the ATP dataset. Interquartile range (IQR) of boxplots represent the 25th and 75th percentiles while maxima and minima represent the maximum and minimum values outside 1.5 times the IQR. The central line represents the median within that group.

We next decided to investigate if compositional changes within the oral microbiome are present before the diagnosis of breast, prostate, and colon cancer. Like our retrospective analysis we first examined changes in overall microbial community structure by looking for differences in alpha and beta diversity between cancer cases and matched non-cancer controls. We found no significant differences in alpha diversity in either cohort in unadjusted, partially adjusted or fully adjusted linear models comparing case vs. control in any of the four metrics (Fig. 4, Supplementary Figs. 11–12, Table 2). Correspondingly we did not find any significant differences in four different beta diversity metrics (weighted UniFrac, unweighted UniFrac, Bray-Curtis dissimilarity, robust Aitchison’s distance) using unadjusted, partially adjusted or fully adjusted PERMANOVA tests (Fig. 4. Supplementary Figs. 13–14, Table 3) (*p* > 0.05). Similar non-significant results were also obtained when all four beta diversity metrics were examined using MiRKAT (Table 3).

Like our previous retrospective analysis, we also examined whether the time between sample collection and diagnosis had a major impact on signal within the case samples. Spearman correlations showed no significant relationships in any of the alpha diversity metrics in both cohorts (Supplementary Figs. 15–16) (*p* > 0.05). Furthermore, examining case samples that were collected within 4 years of diagnosis showed non-significant diversity results except for prostate cancer in the Atlantic PATH cohort which showed a significant increase in Faith’s phylogenetic diversity (*p* = 0.025) and richness (*p* = 0.041) in case participants (Supplementary Figs. 17–18).

After examining overall oral microbial community structure through various diversity metrics, we were interested in determining whether there was any evidence of specific ASVs, or genera being associated with disease status. In both prospective cohorts (Atlantic PATH, ATP) we found no genera being associated with disease status, however, we did find a small number of ASVs associated with disease status in both cohorts. In the Atlantic PATH cohort, we found an increase in the relative abundance of an ASV classified as *Alloprevotella rava* in prostate cancer (Supplementary Figs. 19–20). We additionally found a decrease in the relative abundance of an ASV classified as Streptococcus in colon cancer (Supplementary Figs. 19–20). Interestingly, none of these ASVs overlapped with those identified in the ATP dataset. Within the ATP cohort we detected two ASVs being decreased in relative abundance in colon cancer, although the significance of these taxa was mostly driven by outliers within control samples (Supplementary Figs. 21–22). Within this cohort ANCOM-II also detected that the relative abundance of an ASV classified to an uncultured Stomatobaculum to be decreased in prospective breast cancer samples (Supplementary Figs. 21–22).

As with our previous analysis we were interested in applying Random Forest models to each prospective cancer type to help identify whether disease signatures exist within the oral microbiome. Separate models were generated for each cancer type and cohort. Overall, models for breast cancer performed poorly in both cohorts with accuracies similar to random classification in both Atlantic PATH and ATP cohorts (Supplementary Figs. 23–24). Interestingly, in the case of prostate cancer three of four models in the Atlantic PATH cohort performed slightly above random classification with AUCs ranging from 0.602 to 0.665 (Supplementary Fig. 23). However, in the ATP cohort all models performed at or below an AUC of 0.5 (Supplementary Fig. 24), although it should be noted that 95% confidence intervals on these AUCs were large due to small sample sizes (Table 1).

**Fig. 5:**
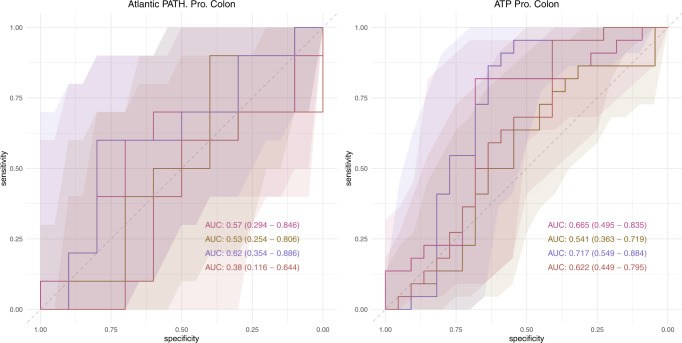
Random Forest Classification performance of prospective cases of colon cancer in the Atlantic PATH and ATP cohorts. Receiver operator curves (ROC) showing the specificity and sensitivity of the classification of non-cancer matched controls and prospective cases of colon cancer in the PATH and ATP datasets. Models were constructed using 100-repeat fivefold cross validation and hold-out performance was determined through taking the mean number of votes for each hold-out sample across all 100 repeats. Within each plot four different ROCs are represented, showing the classification accuracy using ASVs or genus normalized with either total-sum-scaling or center-log-ratio abundance. Shaded areas represent 95% confidence intervals determined through 2000 bootstrap samplings.

Finally, models of prospective cases of colon cancer showed variable results between the two cohorts of interest. With models in the Atlantic PATH cohort showing low performance AUCs ranging from 0.380 to 0.620 (Fig. 5). Contrastingly, in the ATP dataset stronger classification accuracies were found when using center-log-ratio normalizations with the genera level model performing the best (AUC 0.717; 95% CI: 0.549–0.884) (Fig. 5).

**Fig. 6:**
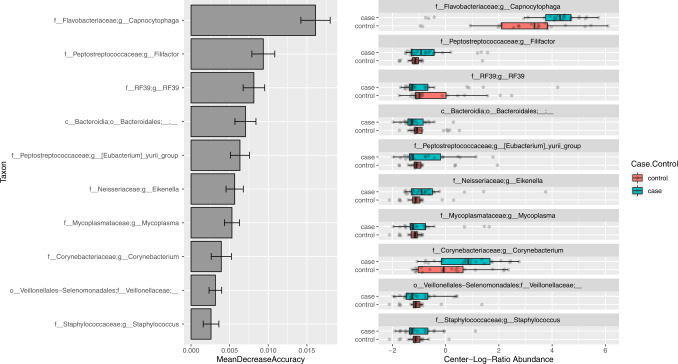
Feature importance was determined using out-of-bag permutation feature analysis. MeanDecreaseAccuracy represents the mean out-of-bag accuracy loss when that feature was randomly permuted across samples and values are shown on the left. Error bars represent standard deviations. Feature center-log-ratio abundance patterns are shown on the right and show possible examples of interesting genera to further investigate in future studies. Interquartile range (IQR) of boxplots represent the 25th and 75th percentiles while maxima and minima represent the maximum and minimum values outside 1.5 times the IQR. The central line represents the median within that group.

Inspecting the top ten most important genera through out-of-bag permutation analysis within our CLR normalized colon cancer model showed no single genera as being particularly important to classification accuracy (accuracy decrease ranging from: 0.003 to 0.016). The most important genera within the model only decreased out-of-bag accuracy by 0.016 (SD; 0.002) although inspection of its CLR abundance did show a notable increase in case samples when compared to non-cancer controls. Inspection of other important genera within this model showed interesting CLR abundance patterns although none were identified in our previous differential abundance analysis (Fig. 6).

Unfortunately, we were unable to validate our prospective models across the two cohorts due to batch effects between them. Indeed, examination of Bray-Curtis dissimilarity profiles of the non-cancer controls between the two cohorts showed significant differences (PERMANOVA *r*^2^: 0.03, *p* = 0.001) most likely caused by different DNA extraction methods (Supplementary Fig. 25). However, we were also interested in examining whether Random Forest models trained on retrospective colon cancer oral microbiome data could accurately classify prospective colon cancer cases within the same cohort. Surprisingly, after training retrospective models showed better than random classification on prospective disease cases (Supplementary Fig. 26). However, as with our previous analysis confidence intervals were large and accuracy was only marginally above random performance.

## Discussion

Herein we examined the oral microbiome in the context of both retrospective and prospective cases of prostate, colon, and breast cancer in a population setting. Our analysis showed no significant changes in oral microbiome diversity in prospective cases of these cancers. Retrospective beta diversity results did show differences in unweighted UniFrac breast cancer profiles. Moreover, weighted UniFrac, Bray-Curtis dissimilarity, and Robust Atchinson’s distance in partially adjusted colon cancer MiRKAT models were also found to be significant. Investigating the relationships of individual taxon and cancer status showed some evidence of potential associations, although the majority were only detected by one of four DA tools tested and showed relatively low effect sizes. Accordingly, Random Forest classification of case samples and non-cancer matched controls showed relatively low classification accuracies with colon cancer showing the strongest signal in both retrospective and prospective analysis. Overall, our findings suggest that no large community changes exist in the oral microbiome of individuals with retrospective or prospective cases of prostate and breast cancer. Although a minor amount of evidence in our report does suggest there may be potential individual taxon relations within these diseases. Contrastingly, signal found in prospective colon cancer cases using Random Forest modelling highlights the need for larger studies on prospective colon cancer cases.

Examining our results in breast cancer more closely showed strong concordance with previous work by Wang et al., who also found no changes in overall oral microbiome composition in United States individuals with breast cancer^29^. These results contrast with a recent study by Wu et al., who identified differences in microbial diversity and the abundance of Porphyromonas and Fusobacterium^30^. This could be due to several reasons including the fact that these studies were conducting under highly different populations, as geographic differences have been shown to impact oral microbiome composition^38^. Unlike either of these studies, we did find evidence for a modest decrease in the relative abundance of Clostridia UCG-014 an uncultured genus that we previously linked to differences in height within healthy individuals of the same cohort^1^. Whether this taxon plays any role in disease status is unclear. Similarly, we also identified an increase in one ASV classified within the genus *Capnocytophaga* which matched with 98% nucleotide identity to an isolate of *Capnocytophaga ochracea* within the Human Oral Microbiome Database. While this taxon hasn’t been linked to cancer other species in this genus such as *C. gingivalis* have previously been associated with oral squamous cell carcinomas^39^.

Investigating signal within the oral microbiome of breast cancer individuals using Random Forest modeling showed relatively little signal with accuracies in retrospective cases only slightly better than random assignment. Based on these modelling results and the above diversity results, we believe it is unlikely that the oral microbiome harbors large shifts in composition that could be used as a breast cancer biomarker in a population setting.

To the best of our knowledge, this report is the first to examine the relationship of the oral microbiome and prostate cancer diagnosis. Like breast cancer, we found no large shifts in oral microbiome diversity in prospective or retrospective cases of prostate cancer. Although, we did see a possible time dependent effect in the prospective Atlantic PATH cohort which was seen in our other prospective cohort. Whether these differing results are due to DNA extraction, regional differences, or simply a false positive discovery would require further investigation in future studies.

Despite not identifying any consistent differences in diversity, multiple ASVs and genera were identified by corncob to be associated with retrospective cases of prostate cancer. Unsurprisingly, comparing these results to those previously identified within the gut showed little overlap^10–12^. In addition, none of these retrospective taxonomic relationships were recovered in our prospective datasets. Although it is interesting to note that some of the identified genera including Actinomyces, Prevotella, Gemella, Granulicatella, Fusobacterium, Neisseria, and Hemophilus were a part of the core genera identified in 99% of individuals in our previous analysis of the oral microbiome in healthy Atlantic Canadians^1^. However, these associations could be related to other broad lifestyle changes that occur after prostate cancer diagnosis. Indeed, several of these retrospective taxa associations were previously associated with various lifestyle, dietary and anthropometric measurements within healthy PATH participants^1^.

Accompanying these results, we saw little to no signal in our Random Forest prostate cancer classification models. Some signal was recovered from the best models trained on the prospective Atlantic PATH cohort (AUROC 0.665); however, due to small sample sizes (*N* = 28) confidence intervals remained large. Moreover, this signal was not recovered in our additional prospective ATP cohort. These results suggest that the oral microbiome is unlikely to be a strong biomarker of prostate cancer risk.

Our colon cancer analysis of oral microbiome diversity only showed significant differences in retrospective cases after the use of partially adjusted MiRKAT models. Other beta diversity testing in both retrospective and prospective cases showed results close to our crital alpha value but remained insignificant. Although this may be attributed to the small size of our datasets. Indeed, these insignificant results conflict with previous work by both Flemer et al., and Wang et al., who found significant differences in oral microbiome beta diversity between healthy controls and individuals diagnosed with colon cancer^4,33^. However, after filtering retrospective sampling to be within 6 years of diagnosis we did find a significant reduction in oral microbiome richness suggesting that time of diagnosis plays an important role in oral microbiome composition. Highlighting the need for future prospective studies on the oral microbiome and colon cancer diagnosis.

In retrospective cases of colon cancer, we found evidence of an increase in an ASV classified as *Fusobacterium peridonticum* by two different microbiome DA frameworks (corncob, ANCOM-II). This species a member of the core oral microbiome genus Fusobacterium has previously been identified as being increased in relative abundance in the oral microbiome of oral small cell carcinoma^40^, head and neck cancer^41^, and pancreatic cancer patients^42^. Whether this oral microbe represents a specific connection to colon cancer, cancer development, or other events associated with cancer diagnosis such as treatment or lifestyle changes is yet to be investigated.

Finally, of the three cancers examined, colon cancer showed the most consistent signal in our Random Forest modeling, although substantial differences in classification accuracies were noted between our two prospective cohorts. This could have been due to a few factors including sample size differences, collection method, or possibly differences in health risk factors between Atlantic Canada and Alberta. Unfortunately, attempts to train Random Forest models on combined datasets were not successful due to different collection and DNA extraction approaches causing significant bias between studies^43,44^.

One puzzling result we noticed from our colon cancer Random Forest models, was that different data normalization had large impacts on model accuracies and was not consistent between retrospective and prospective datasets. We found total-sum-scaling (referred to as relative abundance) to perform the best in our retrospective cohort but found center-log-ratio transformation to perform better in our prospective ATP cohort. Whether this has biological significance is unclear and suggests that future models may be interested in testing several different data normalizations during model training.

Within our analysis we have also identified several limitations that should be noted when reviewing our results. The first is that our study suffered from small sample sizes for both prostate cancer and colon cancer analysis. Specifically, the sample size of prostate cancer case samples was 24 in the retrospective and 28 in the prospective PATH cohort (Table 1). Moreover, the number of colon cancer cases was only 11 in retrospective and 10 in prospective PATH cohort datasets and 22 in the ATP prospective dataset (Table 1). These smaller sample sizes most likely interfered with our ability to detect small differences in both community composition and individual taxa abundances. Furthermore, they also limited our ability to examine the relation between diagnosis time and oral microbiome community shifts. Indeed future retrospective and prospective population studies could benefit from surveying larger populations and making attempts to increase sample size through the use of multiple cohorts. However, pre-planning of how samples will be collected and stored in each of these cohorts is critical. This is because the second limitation we ran into during our study is that different DNA extraction methods were used within the Atlantic PATH and ATP cohorts. Moreover, since these studies were note originally designed to examine the oral microbiome, they suffered from other technical variations such as differences in sample collection and storage. These variations led to the inability to validate machine learning models across cohorts, a critical step in determining model performance on outside datasets. Another side effect of not including oral microbiome analysis in the original intent of these studies led to the inability for us to control for some co-variates that have shown association with microbiome structure including the time of day samples were collected and the individuals oral health.

Finally, we would also like to acknowledge that our datasets were relatively homogenous, being predominantly from white Canadians, with income and education levels above average Canadian census data^45,46^. As such, this significantly limits our ability to identify distinct oral microbial signatures in groups that are disproportionately affected by cancer development. Future work could aim to address these issues by promoting enrollment across a diverse population background and providing additional support to underserved communities when needed.

In conclusion, we believe that our report shows that the oral microbiome is unlikely to be an effective population-based risk marker for cases of prostate or breast cancer, although changes in specific bacterial abundances within these diseases may still exist. Contrastingly, in the case of colon cancer our work indicates that disease status is likely related to changes in the oral microbiome and may be useful as a risk marker for colon cancer development. Future studies should aim to evaluate when oral microbiome changes occur in prospective colon cancer cases to determine its suitability for risk stratification.

## Methods

### Study design

This report includes the analysis of saliva samples from individuals who had previously been enrolled in two regional cohorts within the Canadian Partnership for Tomorrow’s Health project (CanPath), a pan-Canadian prospective cohort study focused on examining the influence of genetics, the environment, and lifestyle factors on Canadian’s health. The regional cohorts of interest for this study include Atlantic PATH (which includes participants from the 4 Atlantic provinces: (Nova Scotia, New Brunswick, Prince Edward Island, and Newfoundland and Labrador) and ATP (participants from the western province of Alberta). For this study, both retrospective (cases diagnosed prior to baseline data and sample collection) and prospective (cases diagnosed after baseline data and sample collection) nested case-control designs were employed. This study was granted ethics approval from Dalhousie University Health Sciences Research Ethics Board (REB #2018-4420). All participants provided written informed consent prior to participation.

### Atlantic partnership for tomorrow’s health (PATH) cohort characteristics

At baseline, demographics, lifestyle, personal and family medical history were self-reported on questionnaires, and a subset attended assessment centers where physical measurements and biospecimens such as saliva were collected. For more details on baseline characteristics of the Atlantic PATH cohort, an in depth descriptive cohort profile has already been published^46^. Follow-up questionnaire data was collected between 2016 and 2019. All participants provided written informed consent prior to participation.

For the purposes of this study sample selection within the Atlantic cohort was divided into either a retrospective or prospective nested case-control design. Prior cancer diagnosis was determined through baseline questionnaires filled out by each participant. All available breast, prostate, and colon cancer case saliva samples were included in this study, and control samples (non-cancer) were selected to match cases (1:5) by sex, age (±3 years), BMI (±3), and smoking status (current vs. never/former). The retrospective design included 588 saliva samples from the Atlantic PATH biospecimen repository based on case and non-cancer control matches to individuals that had been diagnosed with breast (*n* = 61), prostate (*n* = 23), or colon cancer (*n* = 14) prior to baseline saliva collection.

For the prospective design, new incident cases of cancer were determined through follow-up questionnaire surveys filled out by each participant. A one-to-one case control design was used with non-cancer control samples being matched to case samples based on age (±3 years), sex, and BMI (±3). A minor number of current smokers ranging from 0 to 3.70% depending on cancer status were included in this analysis (Table 1). The prospective design included 230 samples from the Atlantic PATH cohort who had breast (*n* = 67), prostate (*n* = 35), or colon cancer (*n* = 13).

The median length of time between sample collection and cancer diagnosis for each cancer can be found in Table 1 along with other sample characteristics broken down by cancer type, study design, and case control status.

### Alberta’s tomorrow project (ATP) cohort characteristics

Recruitment and baseline data collection took place between 2000 and 2015 with biospecimen collection beginning in 2009. Details on cohort overall characteristics, recruitment, and design have been published^45^. Saliva biospecimens used for this study were collected using Oragene DNA OG-250 kits at study centers. Sample characteristics were recorded using self-reported baseline and follow-up questionnaire data on demographics and health risks which were completed both at study centers and through the mail. New incident cases of cancer were confirmed through linkage to Alberta Cancer Register (ACR). Case and control samples were matched in 1:1 design based on age (±2 years), sex, and smoking status (current, former, never). In total 414 saliva samples were identified from ATP’s biospecimen repository based on non-cancer controls and cases of breast (*n* = 102), prostate (*n* = 76), or colon cancer (*n* = 29) that were diagnosed after saliva sample collection. The median length of time between saliva collection and cancer diagnosis along with other relative metadata can be found in Table 1.

### Oral microbiome 16S rRNA gene sequencing

Samples from the Atlantic PATH cohort used both previously characterized^1^ and new to this study cancer case samples. Both previously characterized and novel samples to this study were sequenced and processed at the same time following the same protocol. Frozen saliva samples collected from participants and placed in cryotubes were stored at −80°C and then thawed at room temperature and aliquoted into 96 well plates. In a biosafety cabinet using ethanol for sterilization and nitrile gloves (standard sterile techniques), DNA was extracted using a QIAamp 96 PowerFecal QIAcube HT kit following the manufacturer’s instructions using a TissueLyser II and the addition of Proteinase K at the indicated optional step. Sequencing was done at the Integrated Microbiome Resource at Dalhousie University. PCR amplification of the V4-V5 16 S rRNA gene region was done using V4-V5 fusion primers (515FB - 926 R) and a high-fidelity Phusion polymerase. A total of 25 cycles of PCR were done: denaturing at 98 °C, annealing at 55 °C, and elongating at 72 °C. Pooled sequencing was then conducted using an Illumina MiSeq to produce 300 bp demultiplexed paired-end reads.

This primer set was chosen due to its usage in our previous study examining the relation between the oral microbiome and anthropometric, dietary, and demographic features. Furthermore, this primer set has been previously validated to target a large range of prokaryotes found within the human microbiome^47^. Although it should be noted that specific taxa may be overrepresented or underrepresented when compared to other primer sets. Indeed, no primer set can achieve equal detection efficiency between all microbial members within a sample (Nearing et al., 2021).

Saliva samples from the Alberta’s Tomorrow Project cohort were collected using an Oragene® DNA OG-250 kit manufactured by DNA Genotek. Samples were collected either in person at local study centers or saliva sample kits were sent to participants by regular postal mail with a return envelope included. Participants were instructed not to eat, chew gum, or smoke 30 min prior to providing a saliva sample. They were asked to spit into the container until the saliva reached the indicated level, screw the cap on, shake for 10 s and send the sample back through the mail. DNA from samples were then extracted using a DNA Genotek PrepIT PT-LP2 kit. After extraction samples were sequenced at the Integrated Microbiome Resource in the same manner as samples from the Atlantic PATH cohort.

### 16S rRNA gene sequence processing

Primers were removed from 16S rRNA reads using cutadapt with default settings^48^. Primer-free reads were stitched using the QIIME2 VSEARCH join-pairs plugin^49,50^ using default settings. Stitched reads were then filtered with default settings using the QIIME2 plugin q-score-joined. Reads were then corrected into amplicon sequence variants using the QIIME2 plugin Deblur with a trim length of 360 bp, and one read set as the minimum number required to pass filtering^51^. For each dataset examined, 0.1% of the mean sample depth was calculated and ASVs below this abundance across all samples within that dataset were removed. This is to keep in line with the potential sequence bleed-through rate of an Illumina MiSeq. ASVs were placed into the Greengenes 13_8 99% reference 16S rRNA tree using the QIIME2 fragment-insertion SEPP plugin^52–54^. Rarefaction curves were generated for each dataset separately and a suitable rarefaction depth of 5000 was chosen for the Atlantic PATH retrospective cohort and 3000 for the Atlantic PATH prospective and ATP prospective cohorts. Rarefied data was used to generate both alpha and beta diversity metrics. Samples below these sequencing depths along with those that had no remaining case or control samples were removed from further analysis. In addition, a single sample in the ATP prospective dataset was removed due to significant contamination during sample preparation. Final case-control sample numbers for each dataset that pass all quality filtering are presented in Table 1. ASVs were then assigned taxonomy using a naive Bayesian QIIME2 classifier trained on the 99% Silva V138 16S rRNA database^55,56^.

### Microbial diversity analysis

Alpha and beta diversity metrics were generated using the QIIME2 command “core-metrics-phylogenetic” with the previously described rarefaction values and phylogenetic tree. Diversity matrices were then exported into R and analyzed between case and control samples for each separate cohort and study design. Alpha diversity between case/control samples were examined using linear models with the inclusion of an “extraction_run” covariate for the Atlantic PATH retrospective samples due to the large number of batch extractions and amount of time passed between sample extractions. Additional alpha diversity models were also examined with partially adjusted models including age and sex, or fully adjusted models including age, sex, height, waist-hip-ratio, and daily vegetable servings. In total four different alpha diversity metrics were investigated: Faith’s phylogenetic diversity, Shannon diversity, evenness, and richness. For diversity analysis a nominal *p* value of 0.05 before FDR correction was chosen as our significance threshold before conducting any statistical analysis. Violin boxplots were generated using ggplot2 while jitter points were added using the R package ggbeeswarm^57^.

Beta diversity metrics were compared using a PERMANOVA test between case samples and case matched control samples for each cancer type within each cohort and study design using the ‘adonis2’ function within the vegan R package^58^. In the case of the Atlantic PATH retrospective data, we included the covariate extraction number due to the large number of different extraction runs and time taken between sample extractions for this dataset. Moreover, two additional adjusted models were examined one that adjusted for age and sex, and the other that adjusted for age, sex, height, waist-hip-ratio and daily vegetable servings. An alpha value of 0.05 was chosen before any statistical testing was conducted. In total four different beta diversity metrics were examined: weighted UniFrac, unweighted UniFrac, Bray-Curtis dissimilarity, and Robust Atchison’s distance. These four beta diversity metrics were visualized using principal coordinate analysis using the function cmdscale within an R programming environment and ggplot2. Ellipses were added to each sample type using the function ‘stat_ellipse()’.

Further statistical analysis of beta diversity results were conducted using microbiome regression kernel association testing with the R package MiRKAT. Significance values (*p* values) were calculated using the Davies method unless sample sizes were below 50, where permutation-based *p* values were generated instead. Omnibus *p* values were examined across all four beta diversity metrics previously tested using PERMANOVA analysis. As with the previous analysis associations that were unadjusted, adjusted for age and sex, and adjusted for age, sex, waist-hip-ratio and daily vegetable servings were examined.

### Microbial differential abundance analysis

Differential abundance analysis was conducted using four different tools developed to analyze microbiome data: ALDEx2^59^, ANCOM-II^60,61^, corncob^62^, and MaAsLin2^63^. These tools range in their consistency and power to detect differences between groupings and should give a broad range on the ability to detect differentially abundant taxa^64^. Each tool was run at both the ASV and genus taxonomic levels. All tools were run comparing taxonomic abundance against case vs. control status. Each tool was run separately for each cancer type, cohort, and study design. During the examination of the Atlantic PATH retrospective dataset, we also included DNA extraction as a covariate due to not all samples being extracted at the same time. Like beta diversity and alpha diversity analysis additional adjusted models for age and sex, and age, sex, height, waist-hip-ratio and daily vegetable servings were also examined. For all tools, taxa that were not found in at least 5% of samples were removed from consideration. Filtered *p* values were then corrected for false discovery using Benjamini–Hochberg correction^65^. A nominal *q* value (FDR corrected *p* value) of <0.05 was chosen to determine significance.

ALDeX2 analysis was run using default settings and general linear models. This includes using a center-log-ratio transformation, and 128 Monte Carlo samplings to generate probability distributions from the observed count data.

ANCOM-II was run using scripts available at: https://github.com/FrederickHuangLin/ANCOM-Code-Archive. Genus and ASV abundance tables were first processed using the function “feature_table_pre_process”. The main grouping variable of interest during pre-processing and the determination of structural zeros was case vs. controls. A value of 0.05 was used to determine outlier zeros and outlier values. Pre-processed tables were then passed into the main ANCOM function with the inclusion of DNA extraction batch as a covariate when examining retrospective PATH data. Significance was determined using a percentage cutoff of 70% for the w statistic.

Corncob was run by first importing taxonomic abundance tables and their corresponding metadata into phyloseq objects^66^. The function differentialTest was then run using the above phyloseq object with the “wald” test option. The phi formula was set to match the phi-null formula to control for differences in variability across sample groupings.

MaAsLin2 was run using default settings and an arcsine transformation. Case vs. control was used as a fixed effect. In the case of the PATH retrospective dataset an additional fixed effect of the DNA extraction batch was included.

### Random forest model training

Random Forest models were trained and used to classify case and control samples from each dataset and study design. Training and classification were done using 100-repeat-5-fold cross validation. In the retrospective dataset control samples were randomly downsampled within each fold training session to avoid unbalanced model training and biasing data within the hold-out fold. In all datasets taxon found in <5% prevalence were filtered out prior to model training. After training and cross validation, the mean number of votes on each hold-out set across all repeats was then calculated. Receiver operator curves were constructed using pROC and confidence intervals were estimated using 2000 bootstrap replicates^67^. Variable importance was calculated from models trained on the entire dataset by determining the difference in the out-of-bag prediction error rate after the variable of interest was permuted.

An additional Random Forest model was also trained on the entire retrospective PATH colon cancer dataset using 5-repeat-5-fold cross validation to determine the optimal ‘mtry’ parameter. This trained model was than tested on the prospective PATH dataset to determine model accuracy on future colon cancer diagnosis.

### Reporting summary

Further information on research design is available in the Nature Research Reporting Summary linked to this article.

## Supplementary information

Reporting Summary

Supplemental Figures

## Data Availability

All sequence data has been uploaded to the European Nucleotide Archive and are available under the accession numbers PRJEB38175 and PRJEB56605. Code to analyze all data is available on GitHub at https://github.com/nearinj/Oral_Microbiome_Prostate_Breast_Colon_Cancer. A subset of deidentified metadata used in this project can also be found at the above GitHub link. Additional metadata variables and access to remaining saliva samples can be obtained by contacting either the Atlantic Partnership for Tomorrow’s Health project or the Alberta’s Tomorrow Project.
