# Supplementary material for: Investigating the oral microbiome in retrospective and prospective cases of prostate, colon, and breast cancer
Source: NPJ Biofilms Microbiomes. 2023 May 1;9:23. doi: 10.1038/s41522-023-00391-7 (PMC10151362; doi:10.1038/s41522-023-00391-7)
Supplement: Supplementary file 1 — Reporting Summary [file 41522_2023_391_MOESM1_ESM.pdf]

nature portfolio

Corresponding author(s): Jacob Nearing

Last updated by author(s): Feb 28th 2023

Reporting Summary

Nature Portfolio wishes to improve the reproducibility of the work that we publish. This form provides structure for consistency and transparency in reporting. For further information on Nature Portfolio policies, see our Editorial Policies and the Editorial Policy Checklist.

Statistics

For all statistical analyses, confirm that the following items are present in the figure legend, table legend, main text, or Methods section.

n/a	Confirmed	
☐	☒	The exact sample size (*n*) for each experimental group/condition, given as a discrete number and unit of measurement
☐	☒	A statement on whether measurements were taken from distinct samples or whether the same sample was measured repeatedly
☐	☒	The statistical test(s) used AND whether they are one- or two-sided<br>*Only common tests should be described solely by name; describe more complex techniques in the Methods section.*
☐	☒	A description of all covariates tested
☐	☒	A description of any assumptions or corrections, such as tests of normality and adjustment for multiple comparisons
☐	☒	A full description of the statistical parameters including central tendency (e.g. means) or other basic estimates (e.g. regression coefficient) AND variation (e.g. standard deviation) or associated estimates of uncertainty (e.g. confidence intervals)
☐	☒	For null hypothesis testing, the test statistic (e.g. *F*, *t*, *r*) with confidence intervals, effect sizes, degrees of freedom and *P* value noted<br>*Give P values as exact values whenever suitable.*
☒	☐	For Bayesian analysis, information on the choice of priors and Markov chain Monte Carlo settings
☒	☐	For hierarchical and complex designs, identification of the appropriate level for tests and full reporting of outcomes
☐	☒	Estimates of effect sizes (e.g. Cohen's *d*, Pearson's *r*), indicating how they were calculated

Our web collection on statistics for biologists contains articles on many of the points above.

Software and code

Policy information about availability of computer code

Data collection

The following software was used to analysis this data:
- QIIME2 v2020.8
- "cutadapt" v 1.14
R version 3.6.3 with the following packages:
"ALDEx2" v1.18.0
"exactRankTests" v0.8.31
"nlme" v3.1.149
"dplyr" v0.8.5
"ggplot2" v3.3.0
"compositions" v1.40.2
"corncob" v0.1.0
"phyloseq" v1.29.0
"Maaslin2" v0.99.12
"cowplot" v1.0.0
"vegan" v2.5.6
"ggbeeswarm" v0.6.0

Data analysis

Code to analyze all data is available on GitHub at https://github.com/nearinj/Oral_Microbiome_Prostate_Breast_Colon_Cancer.

For manuscripts utilizing custom algorithms or software that are central to the research but not yet described in published literature, software must be made available to editors and reviewers. We strongly encourage code deposition in a community repository (e.g. GitHub). See the Nature Portfolio guidelines for submitting code & software for further information.

Data

Policy information about availability of data

All manuscripts must include a data availability statement. This statement should provide the following information, where applicable:

- Accession codes, unique identifiers, or web links for publicly available datasets
- A description of any restrictions on data availability
- For clinical datasets or third party data, please ensure that the statement adheres to our policy

All sequence data has been uploaded to the European Nucleotide Archive and are available under the accession numbers PRJEB38175 and PRJEB56605.A subset of de-identified metadata used in this project can also be found at the above GitHub link. Additional metadata variables and access to remaining saliva samples can be obtained by contacting either the Atlantic Partnership for Tomorrow's Health project or the Alberta's Tomorrow Project.

Human research participants

Policy information about studies involving human research participants and Sex and Gender in Research.

Reporting on sex and gender	This manuscript recorded biological sex and was used as a covariate in hypothesis testing through the manuscript.
Population characteristics	For the Atlantic PATH dataset baseline, demographics, lifestyle, personal and family medical history were self-reported on questionnaires, and a subset attended assessment centers where physical measurements and biospecimens such as saliva were collected. For more details on baseline characteristics of the Atlantic PATH cohort, an in depth descriptive cohort profile has been previously published (Sweeney et al., 2017). Follow-up questionnaire data was collected between 2016-2019.<br><br>For the ATP dataset, recruitment and baseline data collection took place between 2000 and 2015 with biospecimen collection beginning in 2009. Details on cohort characteristics, recruitment, and design have been previously published (Ye et al., 2017).
Recruitment	The Atlantic PATH cohort recruitment used a range of outreach activities, including invitations from the Provincial Health Insurance provider (Nova Scotia only), advertising, media coverage, community and workplace events, incentive programmes (e.g. Airmiles) and community champions who encouraged their friends and families to participate. We have noted potential bias due to these recruitment methods in our discussion.<br><br>The ATP recruitment consists of two phases: Phase I (2000-08) using a two-stage telephone random digit dialing method,22 and Phase II (2009-15) using a volunteer sampling method to recruit participants. Detailed information on these phases can be found in: "Ye et al., 2017. Cohort Profile: Alberta's Tomorrow Project. International Journal of Epidemiology".
Ethics oversight	Dalhousie University Health Sciences Research Ethics Board

Note that full information on the approval of the study protocol must also be provided in the manuscript.

Field-specific reporting

Please select the one below that is the best fit for your research. If you are not sure, read the appropriate sections before making your selection.

☒ Life sciences ☐ Behavioural & social sciences ☐ Ecological, evolutionary & environmental sciences

For a reference copy of the document with all sections, see nature.com/documents/nr-reporting-summary-flat.pdf

Life sciences study design

All studies must disclose on these points even when the disclosure is negative.

Sample size	Sample size was determined based on the number of saliva samples available in each cohort and the number of those samples that were classified as being retrospective or prospective cancer samples.
Data exclusions	A single sample in the ATP prospective dataset was removed due to significant contamination during sample preparation.
Replication	We conducted our research in two separate cohorts to determine their overlap and likelihood for replication.
Randomization	We controlled for covarites by matching case samples to non-cancer controls as well as running unadjusted, partially adjusted and fully adjusted models.
Blinding	This study was exploratory in nature and was not blinded.

Reporting for specific materials, systems and methods

We require information from authors about some types of materials, experimental systems and methods used in many studies. Here, indicate whether each material, system or method listed is relevant to your study. If you are not sure if a list item applies to your research, read the appropriate section before selecting a response.

Materials & experimental systems

n/a	Involved in the study
☒	☐ Antibodies
☒	☐ Eukaryotic cell lines
☒	☐ Palaeontology and archaeology
☒	☐ Animals and other organisms
☒	☐ Clinical data
☒	☐ Dual use research of concern

Methods

n/a	Involved in the study
☒	☐ ChIP-seq
☒	☐ Flow cytometry
☒	☐ MRI-based neuroimaging
