# Supplementary material for: Investigating the oral microbiome in retrospective and prospective cases of prostate, colon, and breast cancer
Source: NPJ Biofilms Microbiomes. 2023 May 1;9:23. doi: 10.1038/s41522-023-00391-7 (PMC10151362; doi:10.1038/s41522-023-00391-7)
Supplement: Supplementary file 2 — Supplemental Figures [file 41522_2023_391_MOESM2_ESM.pdf]

Supplemental Figures

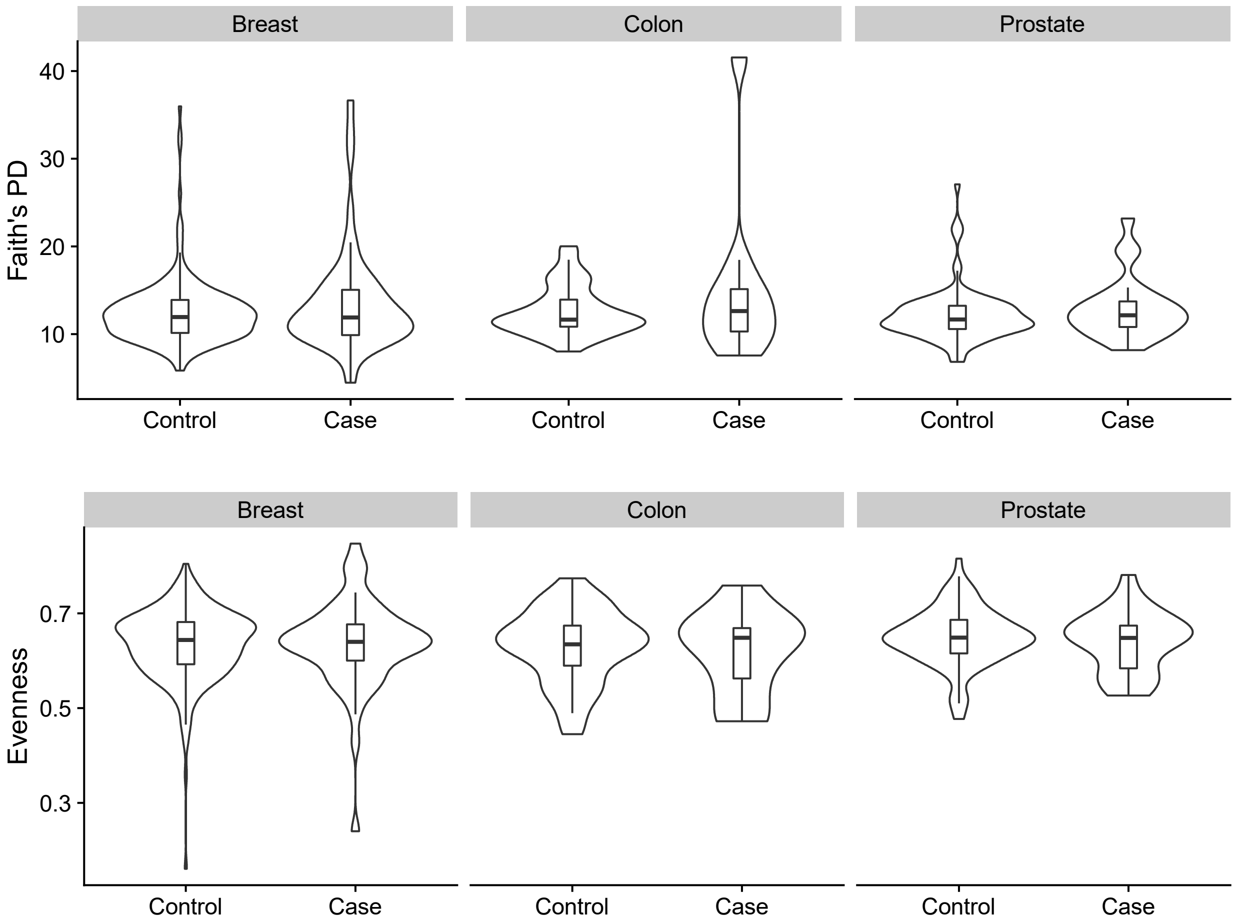

Supplemental Figure 1. Alpha diversity metrics comparing retrospective cases of breast, prostate, and colon cancer to matched controls in the Atlantic PATH cohort. Faith's phylogenetic diversity (top row) and evenness (bottom row) between non-cancer matched controls and retrospective cases of breast, colon, or prostate cancer. We found no significant differences ($p>0.05$) using unadjusted, partially adjusted (age, sex) and fully adjusted (age, sex, height, waist-hip-ratio, daily vegetable servings) linear models. The interquartile range (IQR) of boxplots represent the 25th and 75th percentiles while maxima and minima represent the maximum and minimum values outside 1.5 times the IQR. The central line represents the median within that group.

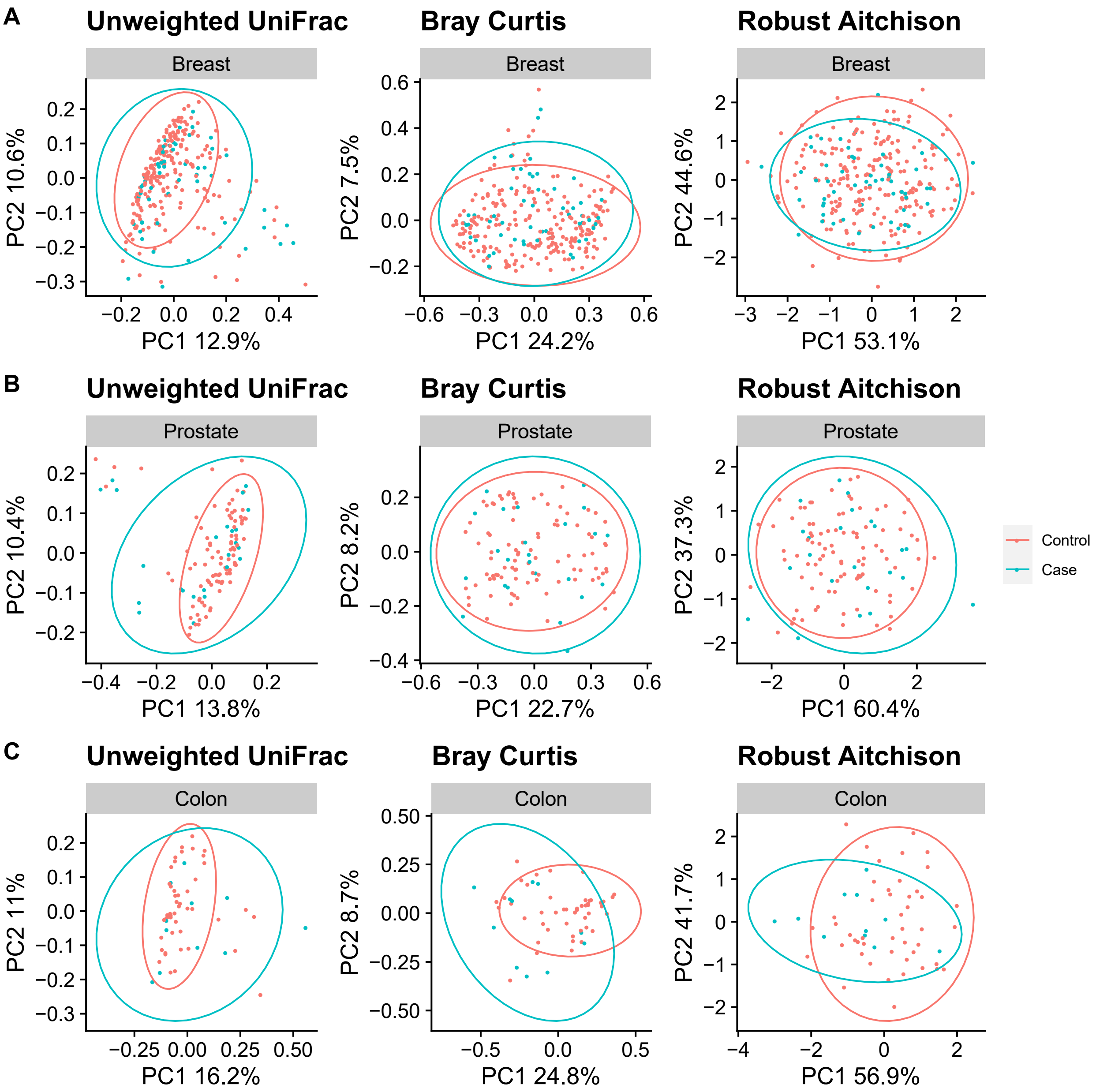

Supplemental Figure 2. Case-Control beta diversity analysis of each cancer type within the retrospective Atlantic PATH cohort. Comparison of three different beta diversity metrics (unweighted UniFrac, Bray-Curtis dissimilarity, and Robust Aitchinson's distance) between non-cancer matched controls and retrospective cases of breast (A) prostate (B) and colon cancer (C). Using unadjusted, partially adjusted or fully adjusted tests resulted in a significant difference in unweighted UniFrac distances in breast cancer case samples. MiRKAT testing showed similar results, although a significant difference in Bray Curtis dissimilarity and Robust Aitchison's distance between colon cancer samples was found after partial model adjustment.

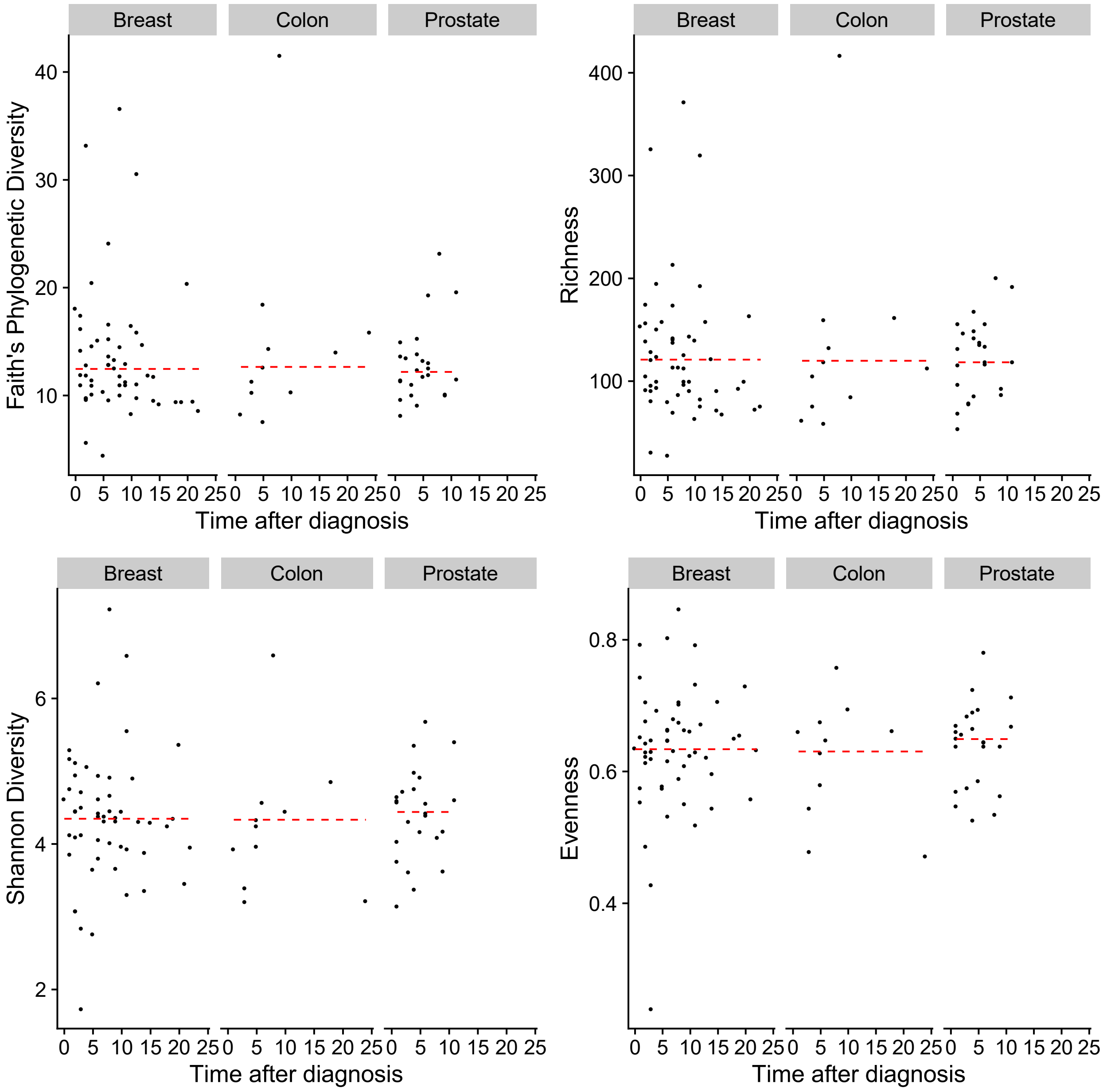

Supplemental Figure 3. Correlation between alpha diversity and time between cancer diagnosis and sample collection in the retrospective Atlantic PATH cohort. Within each retrospective cancer type (breast, colon, and prostate cancer) spearman correlation coefficients were calculated between four different alpha diversity metrics (Faith's phylogenetic diversity, richness, Shannon diversity, evenness) and the time between diagnosis and sample collection. We found a significant positive association between richness and time after diagnosis in colon cancer cases (rho=0.62, p=0.04). Red dotted line represents the mean alpha diversity metric among matched controls.

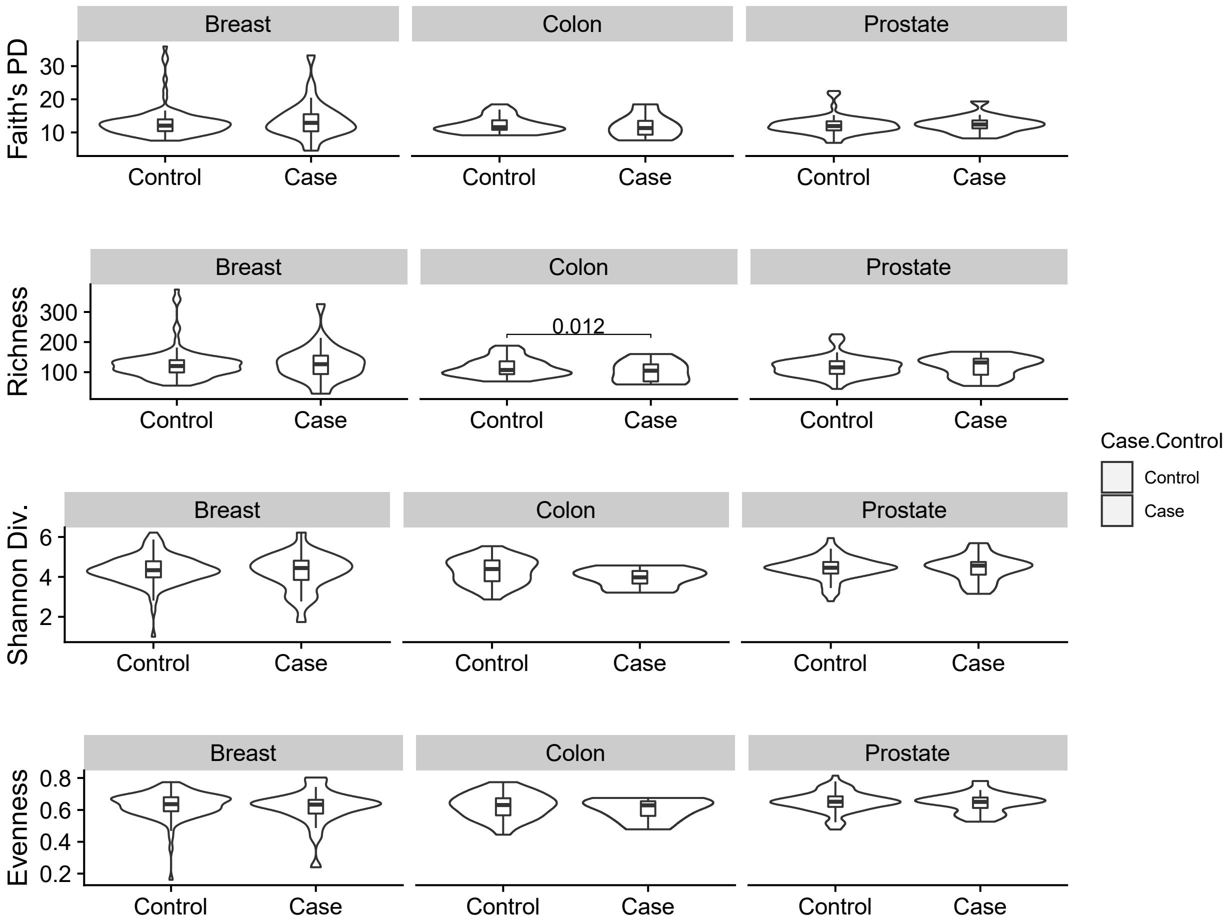

Supplemental Figure 4: **Alpha diversity metrics comparing retrospective cases of breast, prostate, and colon cancer within 6 years to matched controls in the retrospective Atlantic PATH cohort.** Case samples were filtered to only include those that were diagnosed within 6 years of sample collection. Each row of boxplots represents a different alpha diversity metric (Faith's phylogenetic diversity, richness, Shannon diversity, evenness) between non-cancer matched controls and retrospective cases of breast, colon, or prostate cancer. We found a significant difference using linear models while controlling for DNA extraction batch in the richness of retrospective colon cancer cases (p=0.012) this result remained significant after examination using partially and fully adjusted models. Values above bars represent unadjusted p-values. The interquartile range (IQR) of boxplots represent the 25th and 75th percentiles while maxima and minima represent the maximum and minimum values outside 1.5 times the IQR. The central line represents the median within that group.

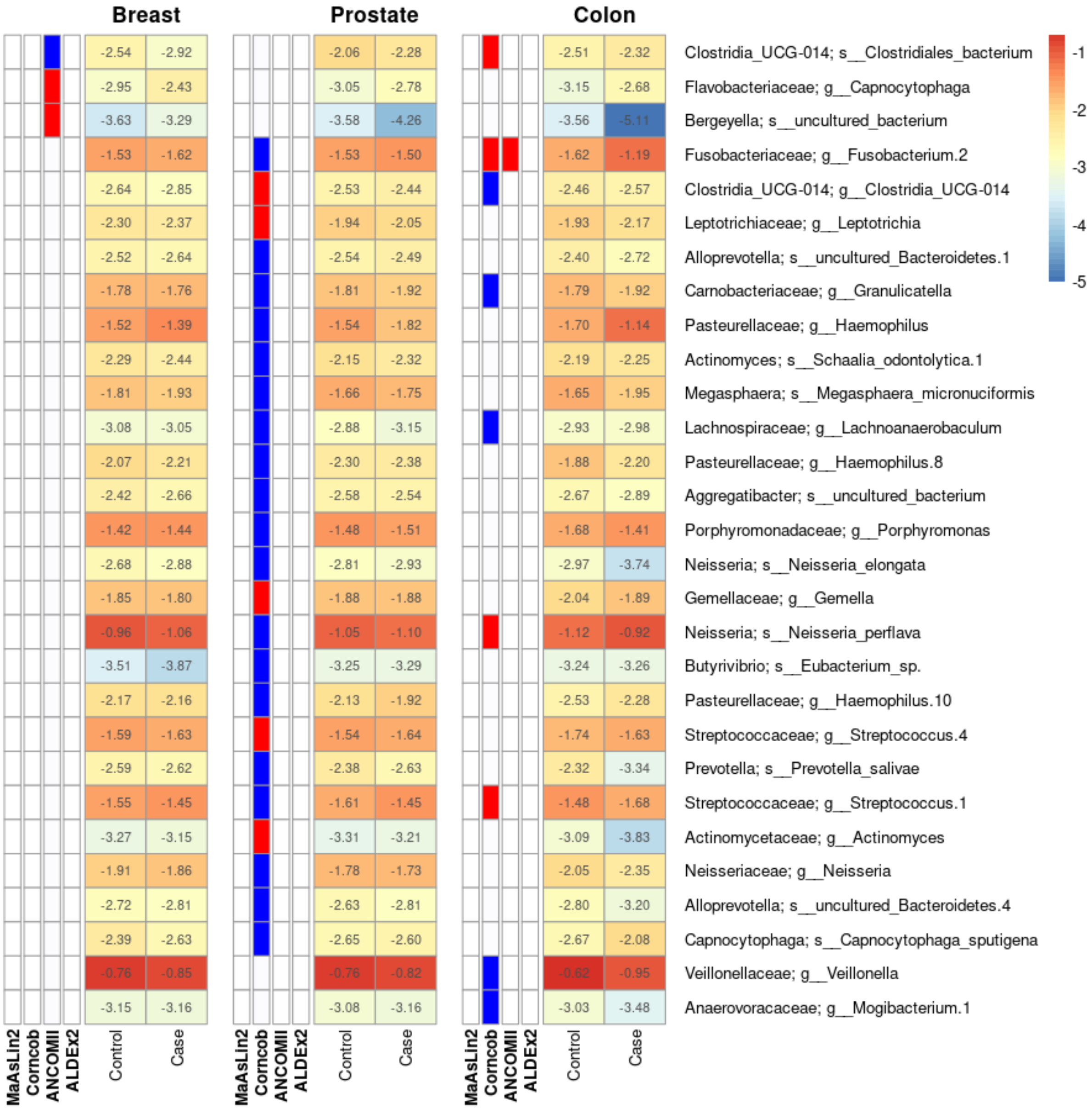

Supplemental Figure 5. Multiple ASV's are differentially abundant in the oral microbiome of retrospective cases of breast, prostate, and colon cancer in the Atlantic PATH cohort. The heatmap is divided by cancer type where the first four columns represent the detection of significant associations by one of four tools: MaAsLin2, Corncob, ANCOM-II, and ALDEx2. Blue bars in the first four columns of each subgroup represent a detected increase in control samples while red bars represent a detected increase in case samples. The final two columns within each cancer sub grouping represent the log10 mean relative abundance of each ASV with red representing higher abundance values and blue representing lower abundance values.

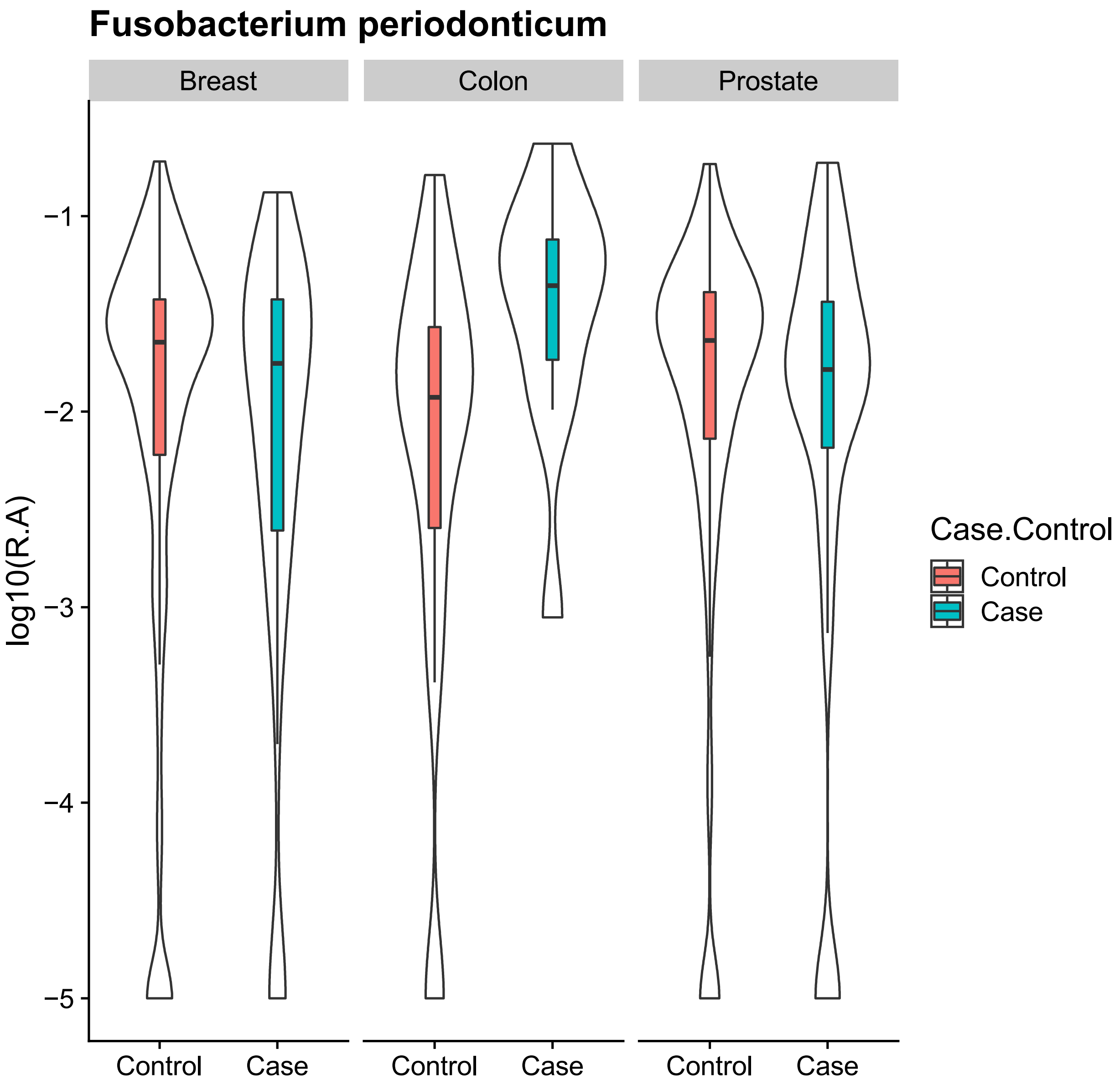

Supplemental Figure 6. An ASV classified as *Fusobacterium periodonticum* is increased in relative abundance within the oral microbiome of retrospective colon cancer samples in the Atlantic PATH cohort. Log10 relative abundances of an ASV best classified as *Fusobacterium periodonticum* across retrospective cases of breast, prostate, and colon cancer. A pseudocount of 0.0001 was added before taking log10 of each sample's relative abundance estimate. The interquartile range (IQR) of boxplots represent the 25th and 75th percentiles while maxima and minima represent the maximum and minimum values outside 1.5 times the IQR. The central line represents the median within that group.

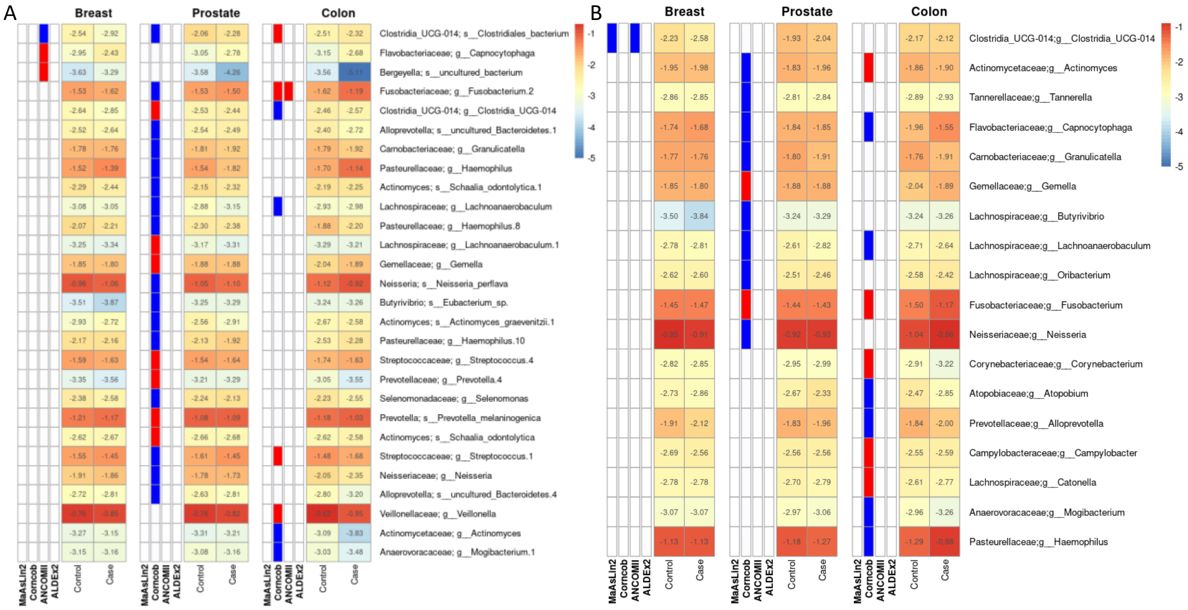

Supplemental Figure 7: Multiple ASV's and Genera are associated with retrospective cancer cases after controlling for participant age and sex. The heatmap is divided by cancer type where the first four columns represent the detection of significant associations by one of four tools: MaAsLin2, Corncob, ANCOM-II, and ALDEx2. Blue bars in the first four columns of each subgroup represent a detected increase in control samples while red bars represent a detected increase in case samples. The final two columns within each cancer sub grouping represent the log10 mean relative abundance of each ASV (A) or genera (B) with red representing higher abundance values and blue representing lower abundance values.

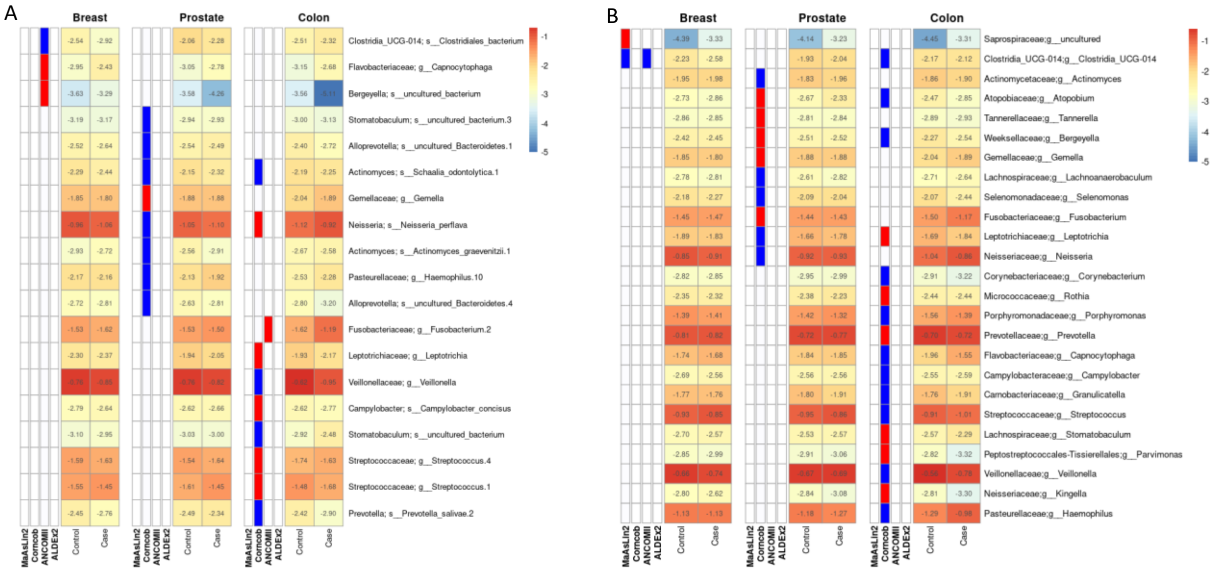

Supplemental Figure 8: Multiple ASV's and Genera are associated with retrospective cancer cases after controlling for participant age, sex, height, waist-hip-ratio, and daily vegetable servings. The heatmap is divided by cancer type where the first four columns represent the detection of significant associations by one of four tools: MaAsLin2, Corncob, ANCOM-II, and ALDEx2. Blue bars in the first four columns of each subgroup represent a detected increase in control samples while red bars represent a detected increase in case samples. The final two columns within each cancer sub grouping represent the log10 mean relative abundance of each ASV (A) or genera (B) with red representing higher abundance values and blue representing lower abundance values.

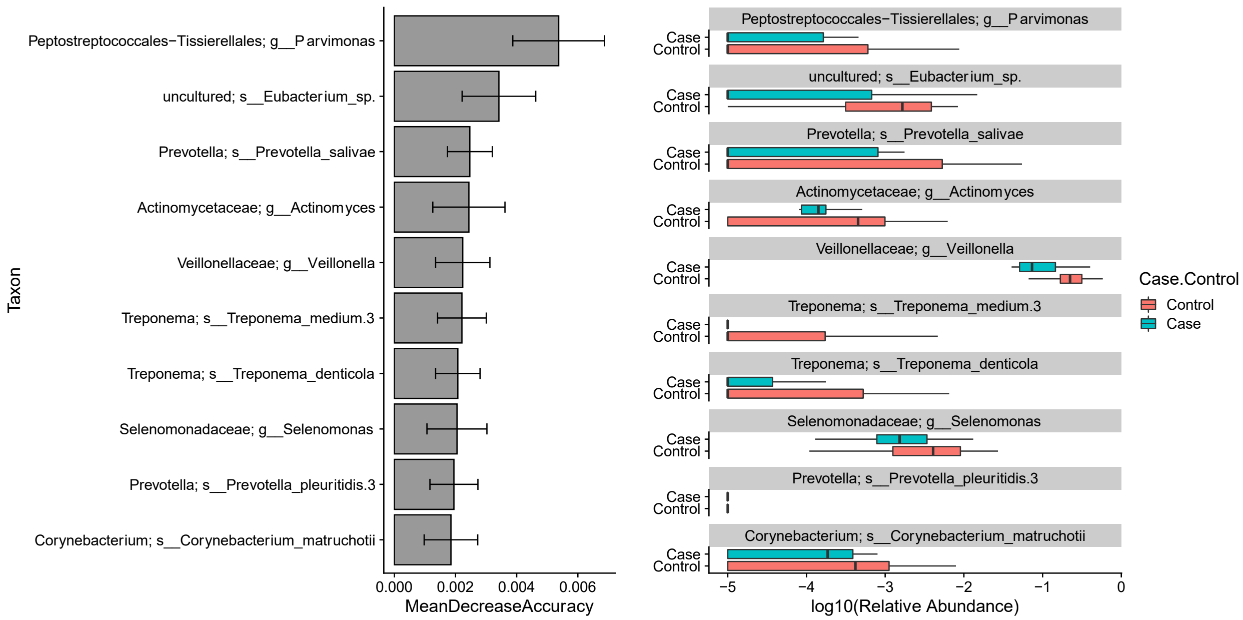

Supplemental Figure 9. Feature Importance in Random Forest classification of retrospective cases of colon cancer using ASVs normalized to relative abundances in the Atlantic PATH cohort. MeanDecreaseAccuracy represents feature importance as determined by comparing out-of-bag accuracies before and after permuting the variable of interest across different samples. Features were then sorted by MeanDecreaseAccuracy and the top 10 were plotted along with their log10 relative abundances. A pseudocount of 0.0001 was added before taking log10 of each sample's relative abundance estimate for each ASV. Error bars on bar plots represent standard deviations. The interquartile range (IQR) of boxplots represent the 25th and 75th percentiles while maxima and minima represent the maximum and minimum values outside 1.5 times the IQR. The central line represents the median within that group.

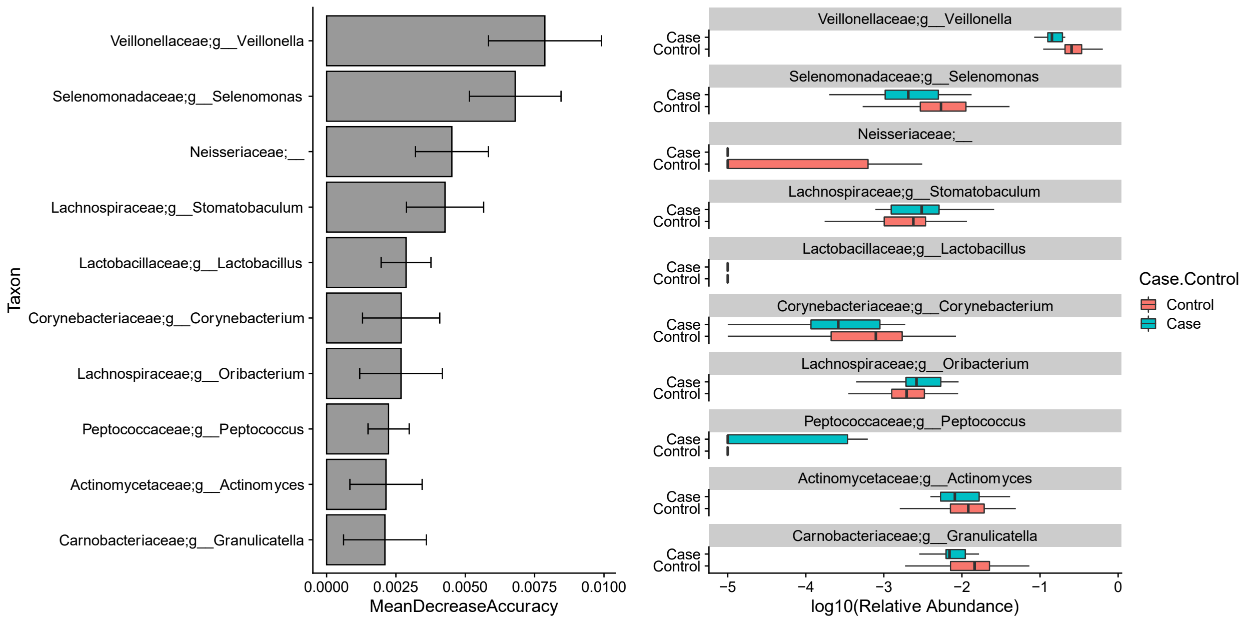

Supplemental Figure 10. Feature Importance in Random Forest classification of retrospective cases of colon cancer using genera normalized to relative abundances in the Atlantic PATH cohort. MeanDecreaseAccuracy represents feature importance as determined by comparing out-of-bag accuracies before and after permuting the variable of interest across different samples. Features were then sorted by MeanDecreaseAccuracy and the top 10 were plotted along with their log10 relative abundances. A pseudocount of 0.0001 was added before taking log10 of each sample's relative abundance estimate for each genus. Error bars on bar plots represent standard deviations. The interquartile range (IQR) of boxplots represent the 25th and 75th percentiles while maxima and minima represent the maximum and minimum values outside 1.5 times the IQR. The central line represents the median within that group.

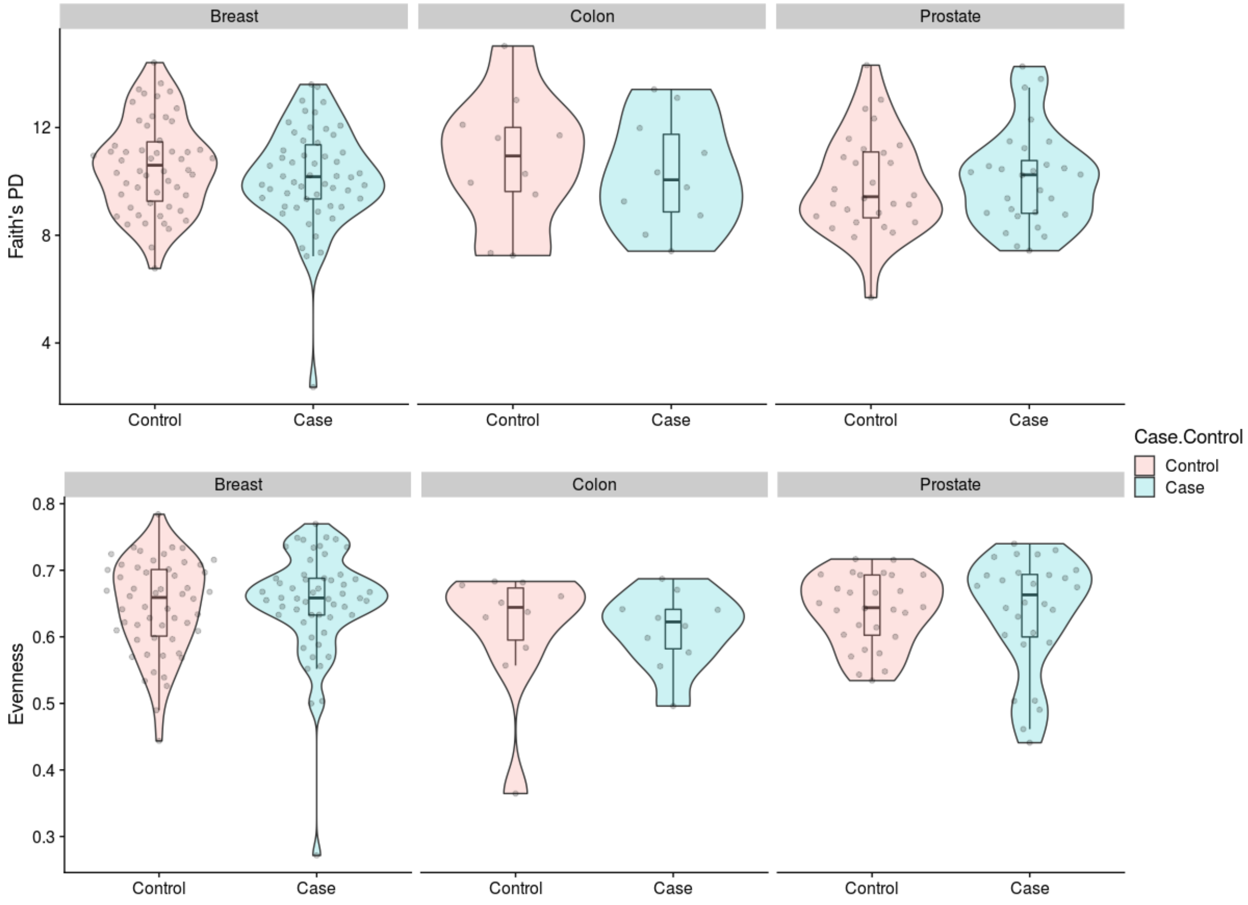

Supplemental Figure 11. Comparison of alpha diversity between prospective cancer cases and non-cancer match controls in the Atlantic PATH cohort. Faith's phylogenetic diversity and evenness were compared within the PATH cohort between non-cancer matched controls and prospective cases of breast, colon, and prostate cancer. Each alpha diversity metric was compared within each cancer type using unadjusted, partially adjusted, or fully adjusted linear models. No significant differences were detected. The interquartile range (IQR) of boxplots represent the 25th and 75th percentiles while maxima and minima represent the maximum and minimum values outside 1.5 times the IQR. The central line represents the median within that group.

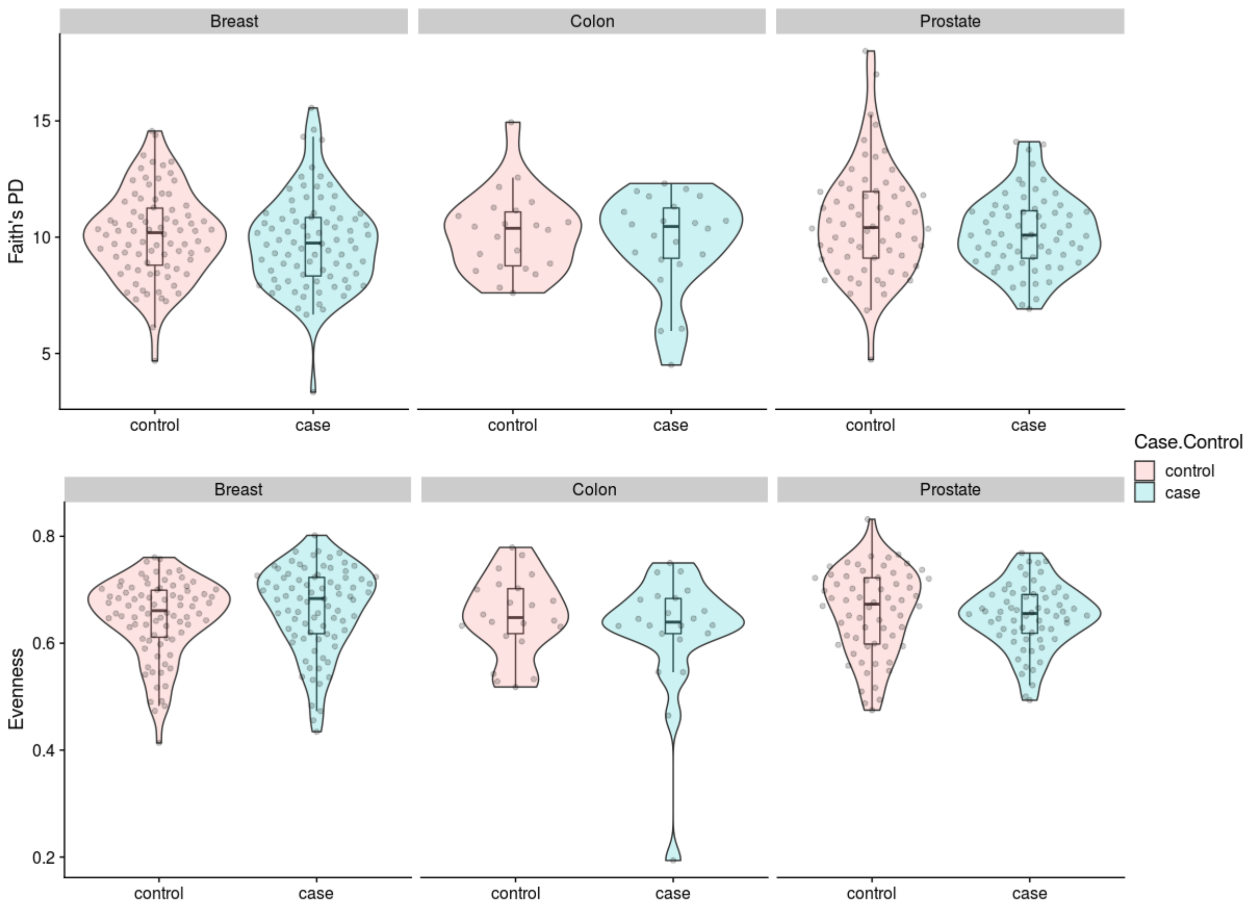

Supplemental Figure 12. Comparison of alpha diversity between prospective cancer cases and non-cancer match controls in the ATP cohort. Faith's phylogenetic diversity and evenness were compared within the ATP cohort between non-cancer matched controls and prospective cases of breast, colon, and prostate cancer. Each alpha diversity metric was compared within each cancer type using unadjusted, partially adjusted, or fully adjusted linear models. No significant differences were detected. The interquartile range (IQR) of boxplots represent the 25th and 75th percentiles while maxima and minima represent the maximum and minimum values outside 1.5 times the IQR. The central line represents the median within that group.

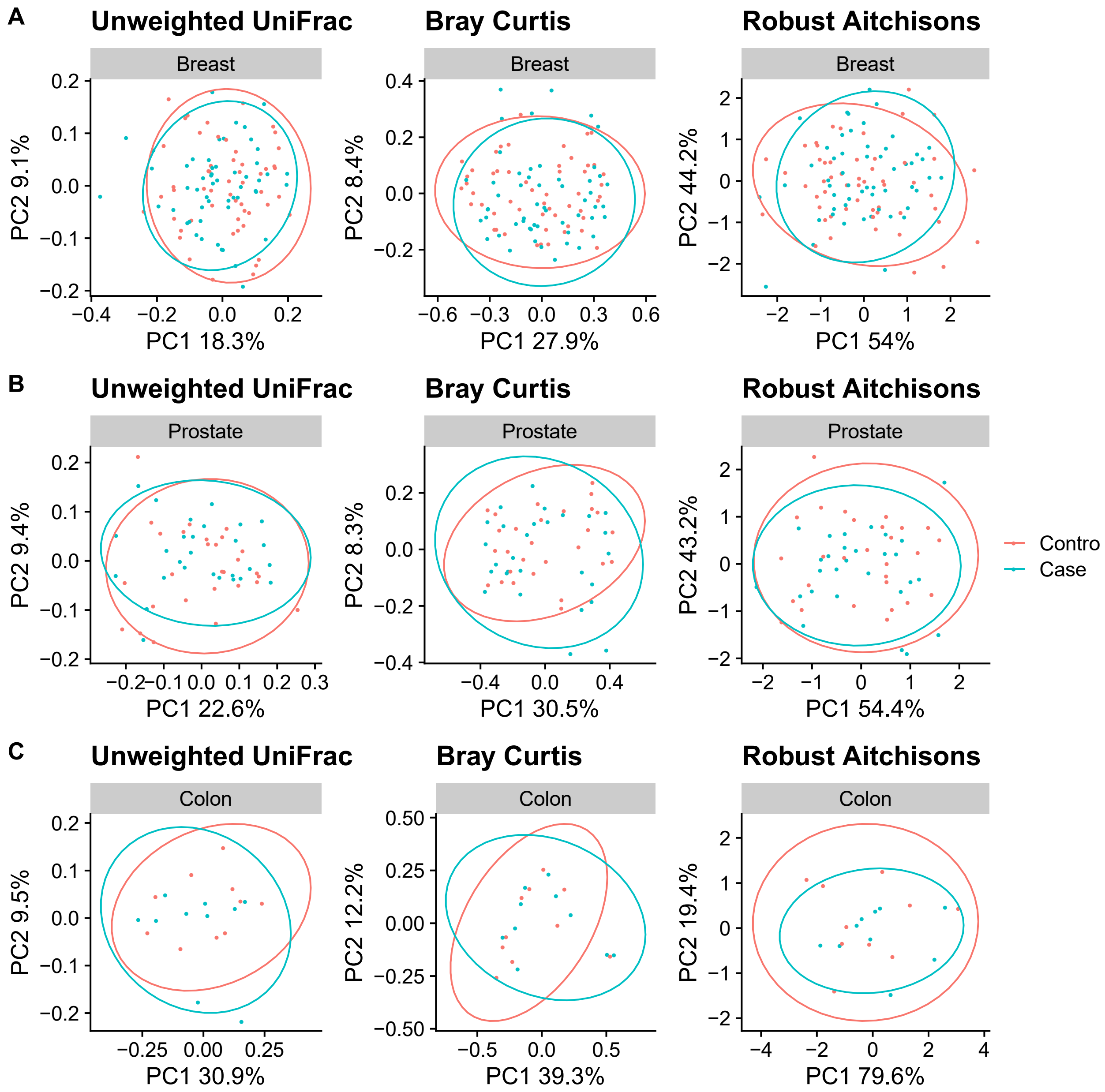

Supplemental Figure 13. Case-Control beta diversity analysis of prospective cases of breast, prostate, or colon cancer in the Atlantic PATH cohort. Three different beta diversity metrics (unweighted UniFrac, Bray-Curtis dissimilarity, and Robust Aitchison's distance) within the PATH cohort were compared between non-cancer matched controls and breast, prostate or colon cancer. No significant differences were found within each metric and cancer type using unadjusted, partially adjusted, or fully adjusted PERMANOVA or MiRKAT tests.

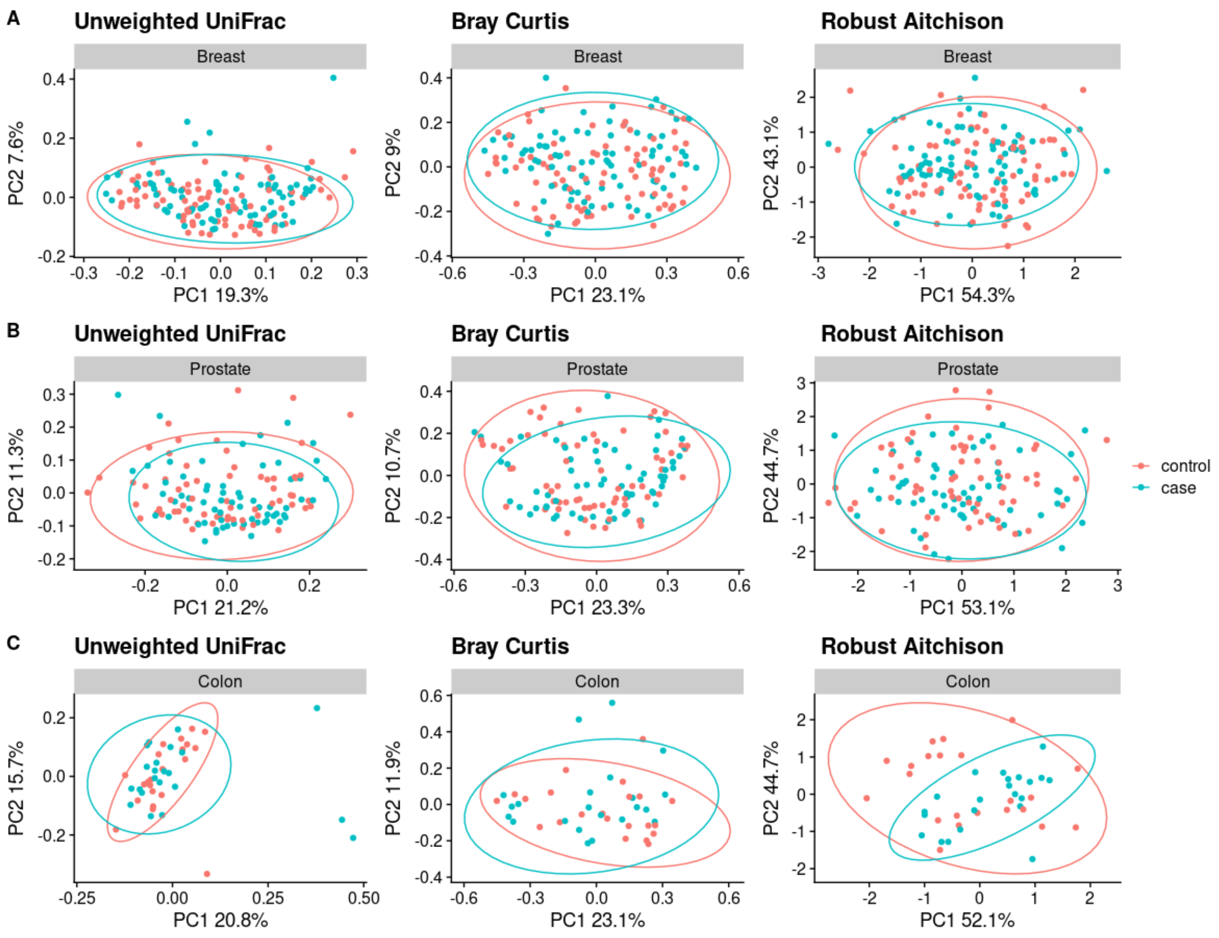

Supplemental Figure 14. Case-Control beta diversity analysis of prospective cases of breast, prostate, or colon cancer in the ATP cohort. Three different beta diversity metrics (unweighted UniFrac, Bray-Curtis dissimilarity, Robust Aitchison's distance) within the ATP cohort were compared between non-cancer matched controls and breast, prostate or colon cancer. No significant differences were found within each metric and cancer type using unadjusted, partially adjusted, or fully adjusted PERMANOVA or MiRKAT tests.

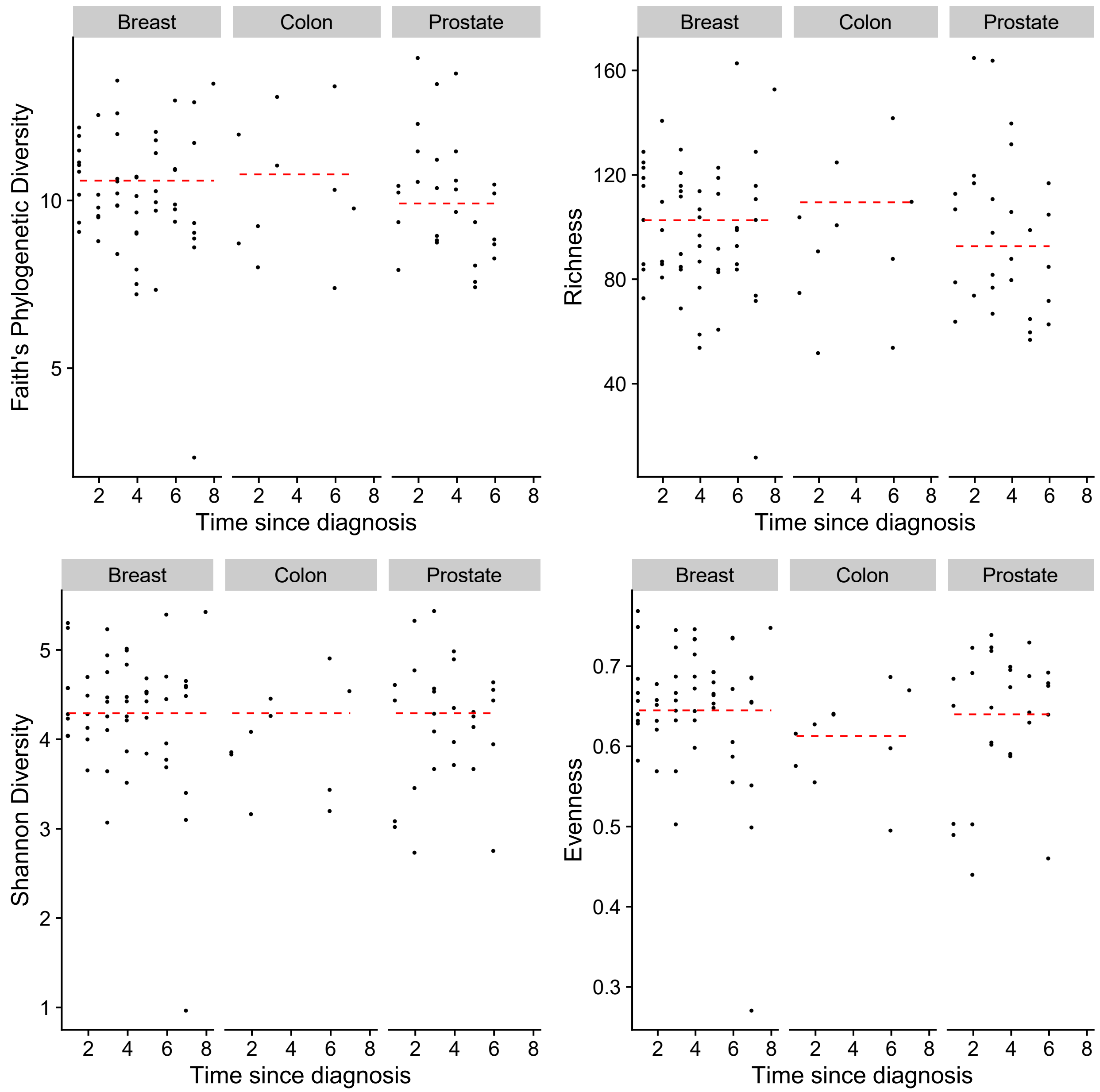

Supplemental figure 15. Correlation between alpha diversity and time since diagnosis in prospective cancer cases within the Atlantic PATH cohort. Within the cohort prospective cancer cases were divided into type (breast, colon, and prostate cancer) and spearman correlations were calculated between four different alpha diversity metrics (Faith's phylogenetic diversity, richness, Shannon diversity, evenness) and the time between sample collection and disease diagnosis. No significant correlations were found. Red dotted line represents mean value in matched controls.

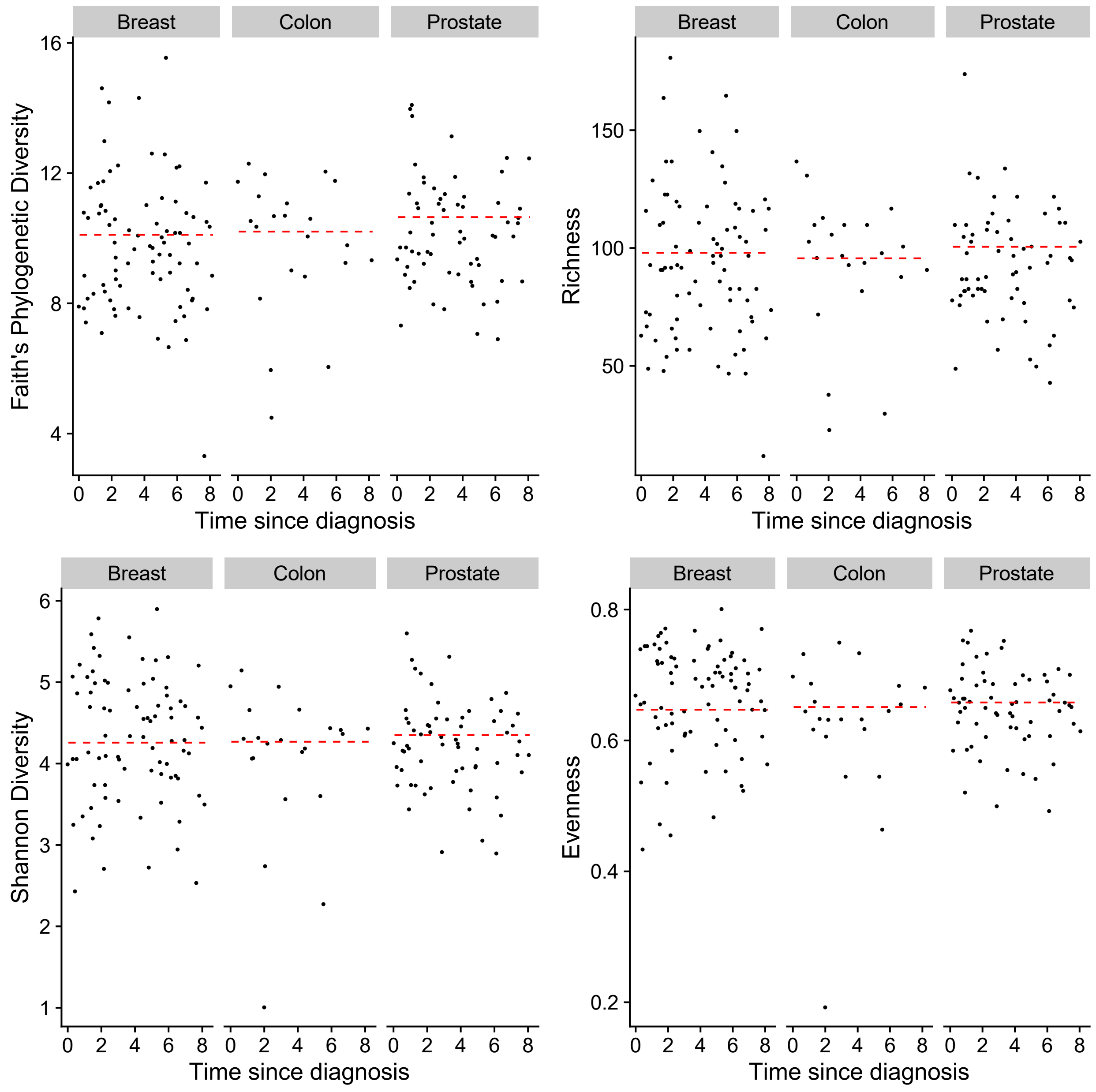

Supplemental Figure 16. Correlation between alpha diversity and time since diagnosis in prospective cancer cases within the ATP cohort. Within this cohort prospective cancer cases were divided into type (breast, colon, and prostate cancer) and spearman correlations were calculated between four different alpha diversity metrics (Faith's phylogenetic diversity, richness, Shannon diversity, evenness) and the time between sample collection and disease diagnosis. No significant correlations were found. Red dotted line represents mean value in matched controls.

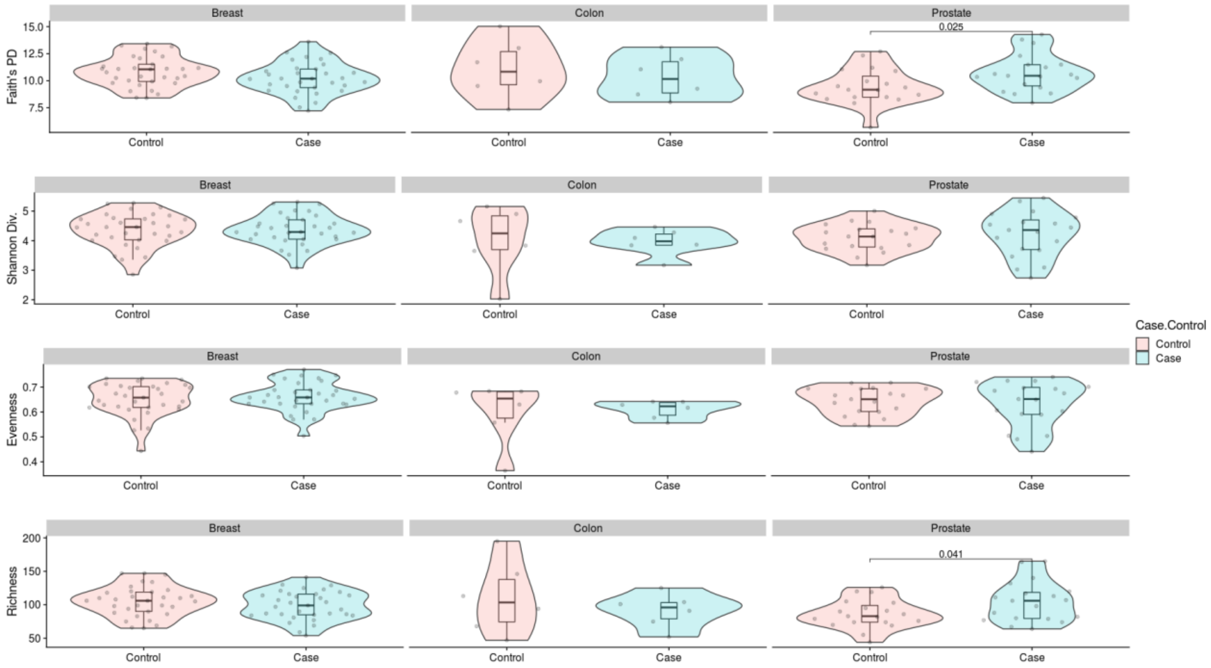

Supplemental Figure 17. Alpha diversity metrics comparing prospective cases of breast, prostate, and colon cancer within 4 years to matched non-cancer controls in the Atlantic PATH cohort. Prospective case samples within the cohort were filtered to only include those that were diagnosed within 4 years of sample collection. Four different alpha diversity metrics (Faith's phylogenetic diversity, richness, Shannon diversity, evenness) were compared between non-cancer matched controls and prospective cases of breast, colon, or prostate cancer. We found a significant difference using unadjusted, partially adjusted or fully adjusted linear models in the richness and unadjusted and partially adjusted models in Faith's phylogenetic diversity for prospective prostate cancer samples. Values above bars present p-values. The interquartile range (IQR) of boxplots represent the 25th and 75th percentiles while maxima and minima represent the maximum and minimum values outside 1.5 times the IQR. The central line represents the median within that group.

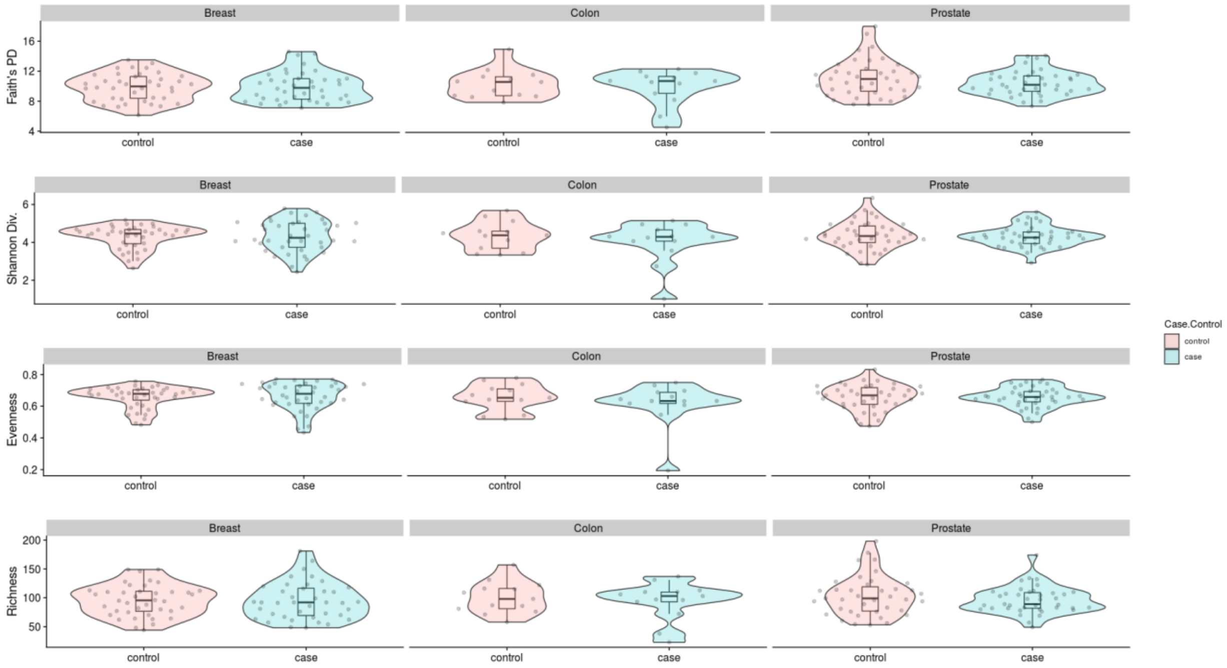

Supplemental Figure 18. Alpha diversity metrics comparing prospective cases of breast, prostate, and colon cancer within 4 years to matched non-cancer controls in the ATP cohort. Prospective case samples with the ATP cohort were filtered to only include those that were diagnosed within 4 years of sample collection. Four different alpha diversity metrics (Faith's phylogenetic diversity, richness, Shannon diversity, evenness) were compared between non-cancer matched controls and prospective cases of breast, colon, or prostate cancer. No significant differences were found using unadjusted, partially adjusted, or fully adjusted linear models comparing case and control samples. The interquartile range (IQR) of boxplots represent the 25th and 75th percentiles while maxima and minima represent the maximum and minimum values outside 1.5 times the IQR. The central line represents the median within that group.

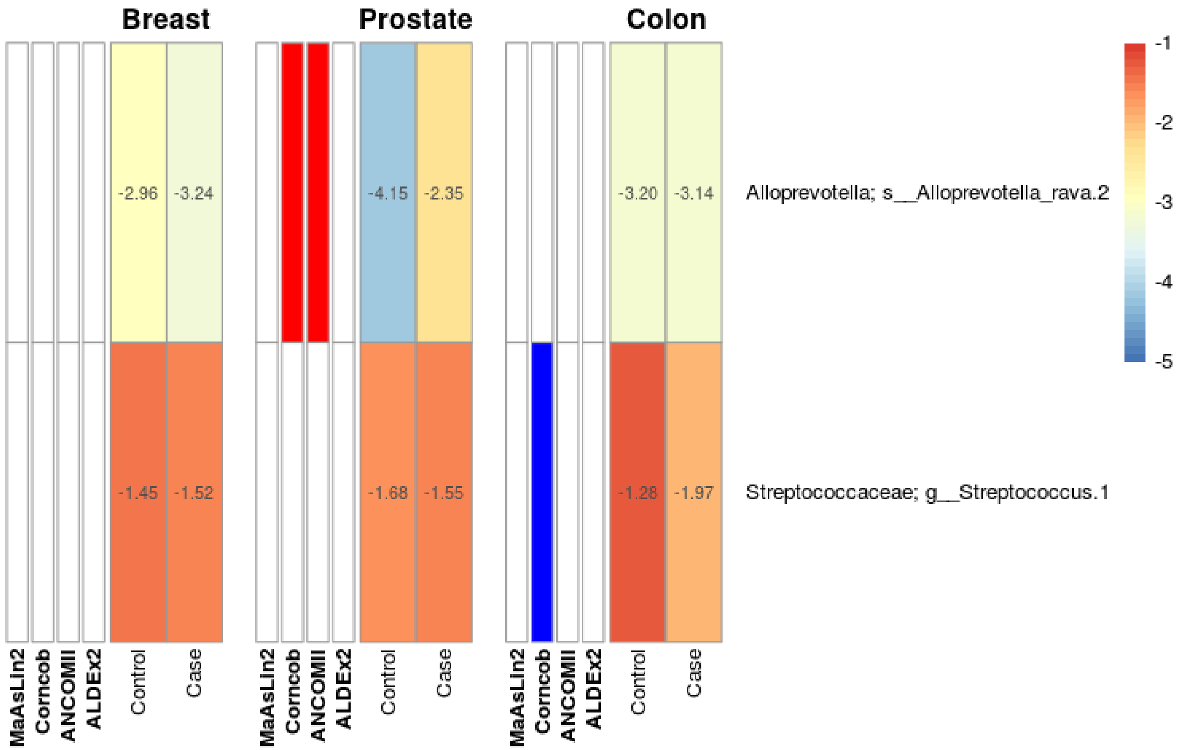

Supplemental Figure 19. Two ASVs were detected as being differentially abundant in the oral microbiome of prospective cases of breast, prostate, and colon cancer in the Atlantic PATH cohort. The heatmap is divided by cancer type where the first four columns represent the detection of significant associations by one of four tools: MaAsLin2, Corncob, ANCOM-II, and ALDEx2. Blue bars in the first four columns of each subgroup represent a detected increase in control samples while red bars represent a detected increase in case samples. The final two columns within each cancer sub grouping represent the log10 mean relative abundance of each ASV with red representing higher relative abundance values and blue representing lower relative abundance values. One ASV, classified as *Alloprevotella rava*, was detected by both corncob and ANCOM-II in prospective prostate cancer cases. Additionally, one ASV, classified at the genus level as Streptococcus, was also detected by corncob in prospective colon cancer cases.

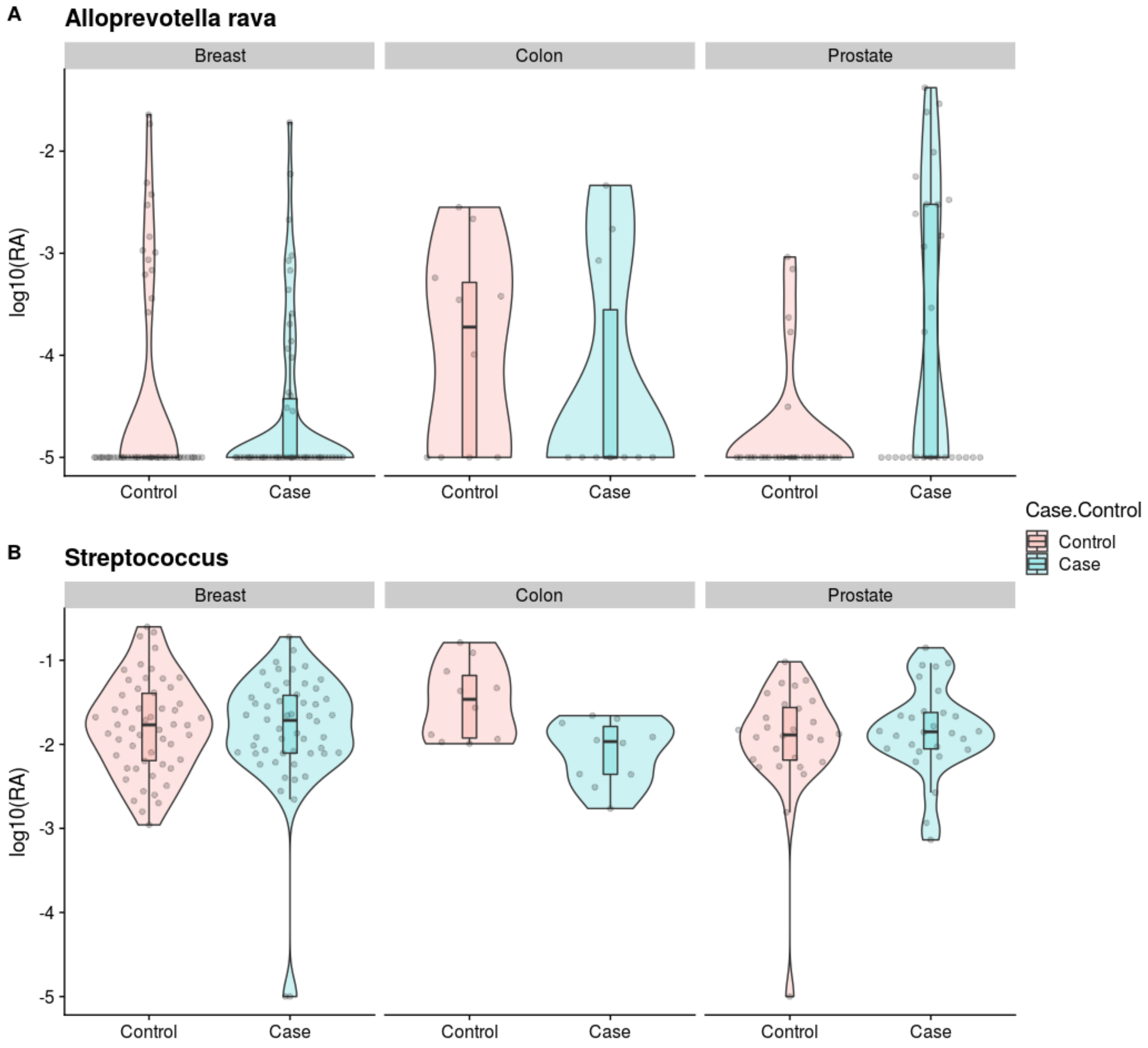

Supplemental Figure 20. ASVs detected as differentially abundant in prospective cases of prostate and colon cancer within the Atlantic PATH cohort. Log10 relative abundance values with a pseudo count of 0.0001 compared between case and control samples for the two ASVs detected as being differentially abundant in prospective cases of prostate cancer (A) and colon cancer (B). The interquartile range (IQR) of boxplots represent the 25th and 75th percentiles while maxima and minima represent the maximum and minimum values outside 1.5 times the IQR. The central line represents the median within that group.

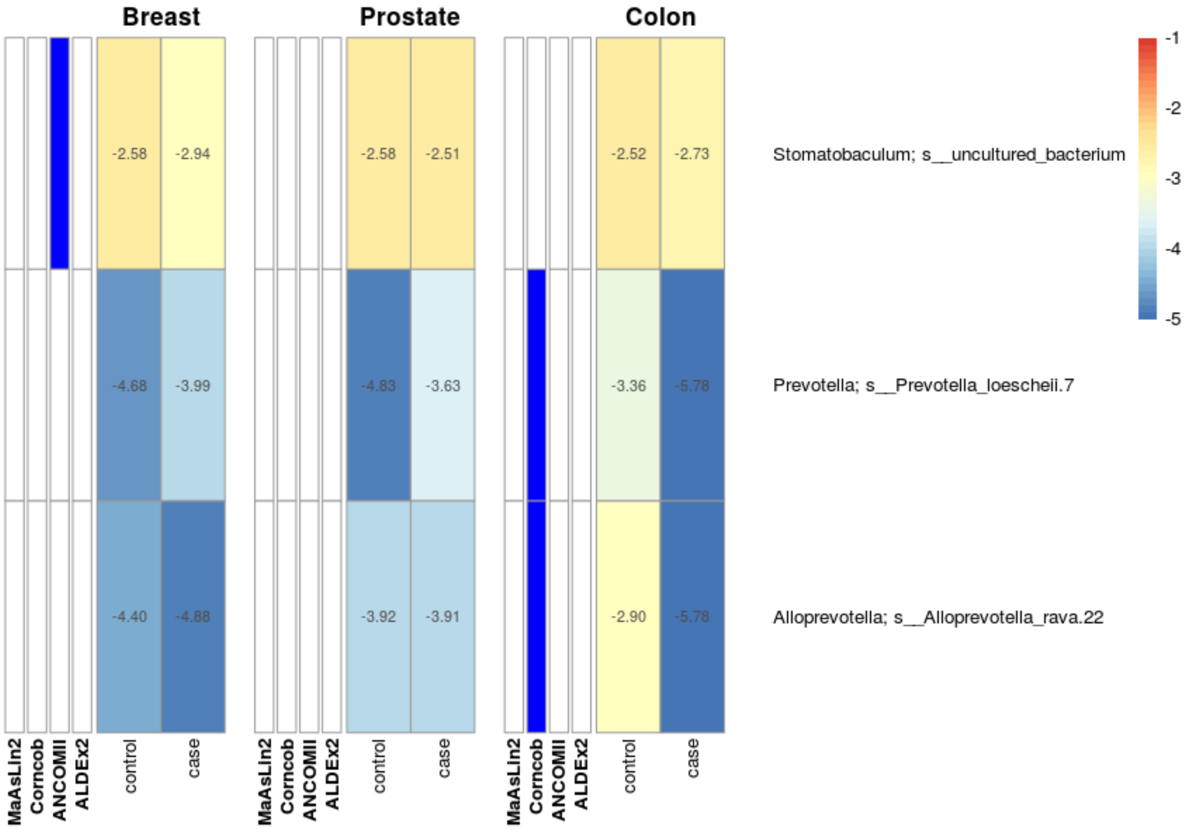

Supplemental Figure 21. Two ASVs were detected as being differentially abundant in the oral microbiome of prospective cases of colon cancer in the ATP cohort. The heatmap is divided by cancer type where the first four columns represent the detection of significant associations by one of four tools: MaAsLin2, Corncob, ANCOM-II, and ALDEx2. Blue bars in the first four columns of each subgroup represent a detected increase in control samples while red bars represent a detected increase in case samples. The final two columns within each cancer sub grouping represent the log10 mean relative abundance of each ASV with red representing higher relative abundance values and blue representing lower relative abundance values. Two ASVs, classified as *Prevotella loescheii* and *Alloprevotella rava* were detected by corncob to be decreased in relative abundance in colon cancer. One ASV classified as an uncultured species of Stomatobaculum was detected by corncob to be decreased in relative abundance in breast cancer.

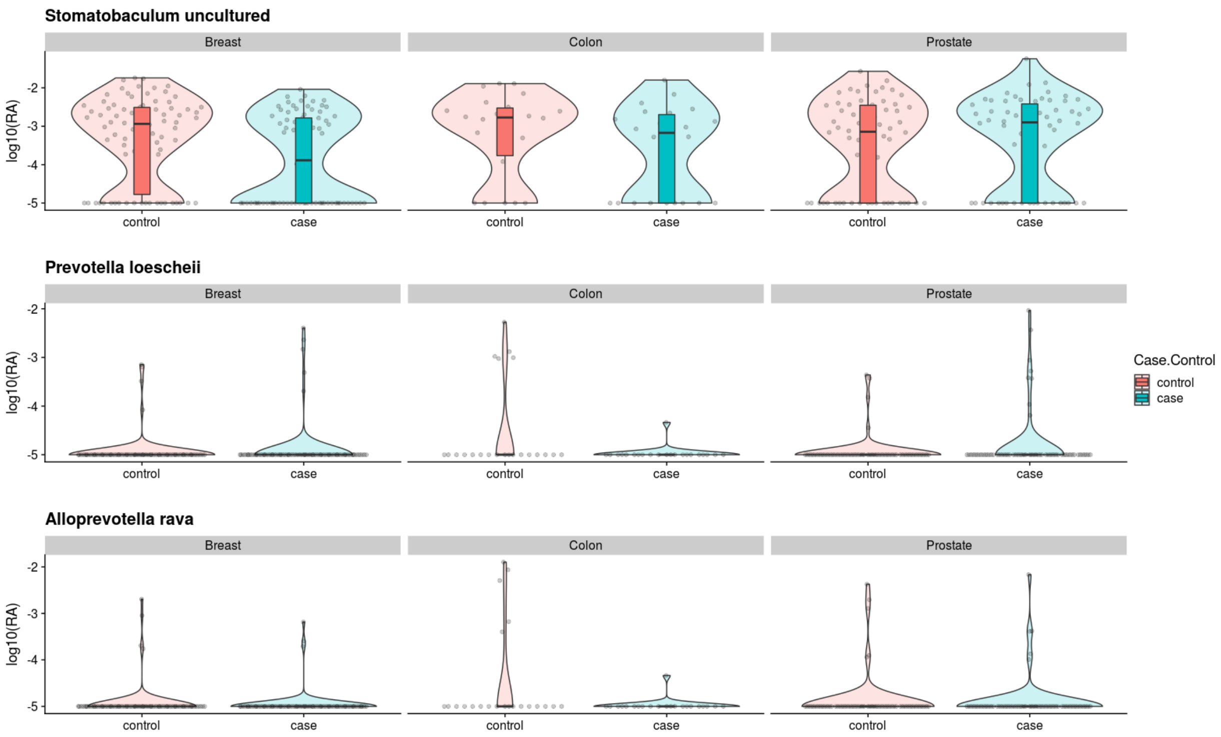

Supplemental Figure 22. ASVs detected as differentially abundant in prospective cases of colon cancer and breast cancer within the ATP cohort. Log10 relative abundance values with a pseudo count of 0.0001 compared between case and control samples for the two ASVs (Prevotella loescheli and Alloprevotella rava) detected as being differentially abundant by corncob in prospective cases of colon cancer. One ASV (Stomatobaculum uncultured) detected as being differentially abundant by corncob in prospective cases of breast cancer. The interquartile range (IQR) of boxplots represent the 25th and 75th percentiles while maxima and minima represent the maximum and minimum values outside 1.5 times the IQR. The central line represents the median within that group.

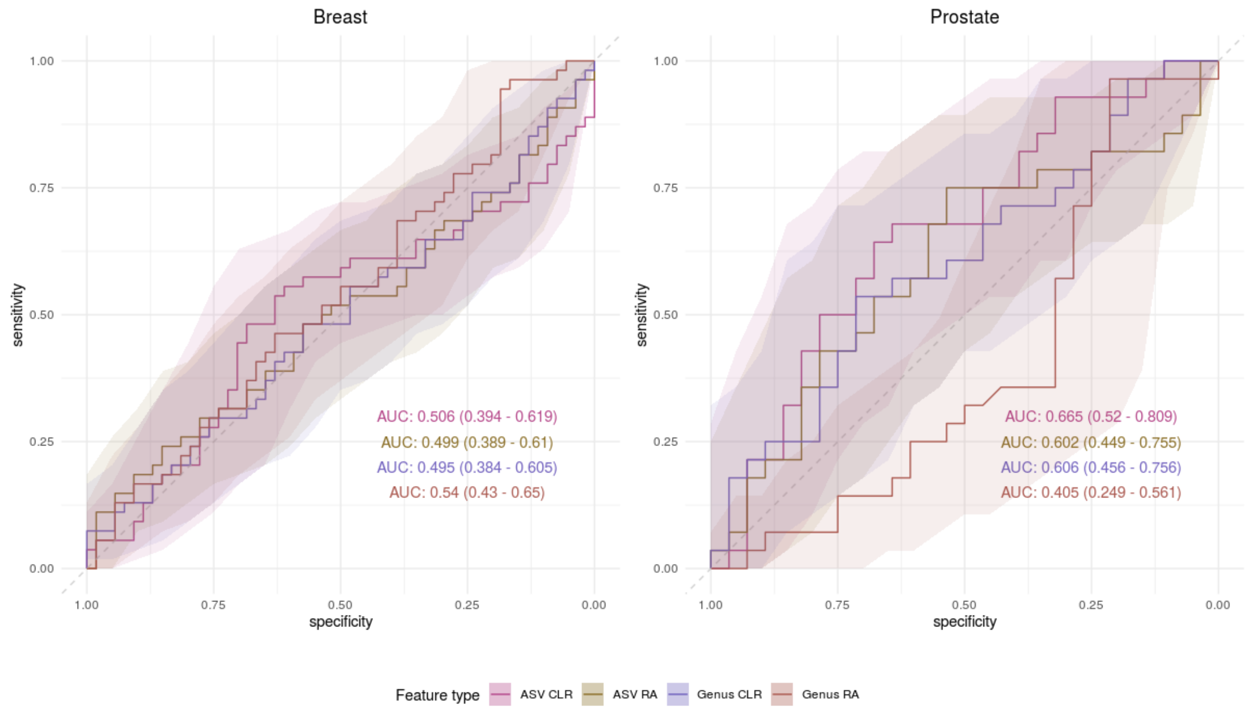

Supplemental Figure 23. Random Forest classification of prospective cases of breast and prostate cancer on microbial taxonomic composition within the Atlantic PATH cohort. Receiver operator curves (ROC) showing the specificity and sensitivity of the classification of non-cancer matched controls and prospective cases of breast, prostate, or colon cancer within the PATH cohort. Models were constructed using 100-repeat 5-fold cross validation and hold-out performance was determined through taking the mean number of votes for each hold-out sample across all 100 repeats. Within each plot four different ROCs are represented showing the classification accuracy using ASVs or genera normalized with either total-sum-scaling or center-log-ratio abundance. Shaded areas represent 95% confidence intervals determined through 2000 bootstrap samplings.

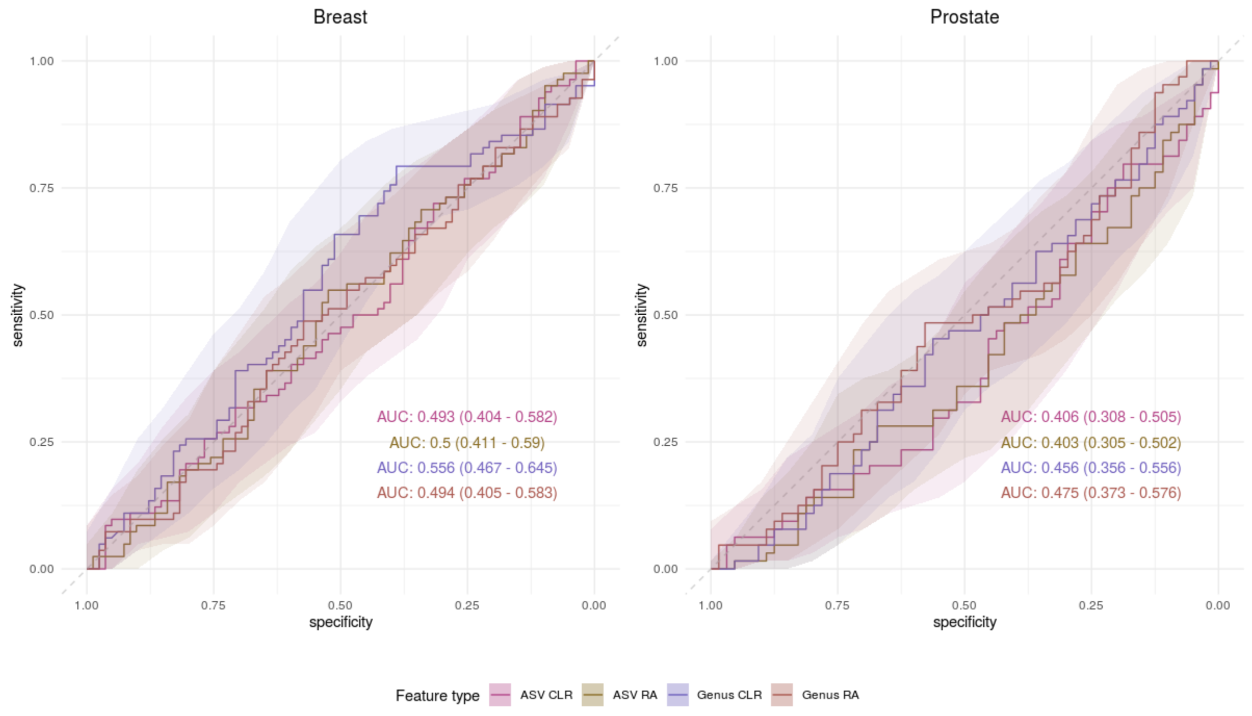

Supplemental Figure 24. Random Forest classification of prospective cases of breast and prostate cancer based on microbial taxonomic composition within the ATP cohort. Receiver operator curves (ROC) showing the specificity and sensitivity of the classification of non-cancer matched controls and prospective cases of breast, prostate, or colon cancer within the ATP cohort. Models were constructed using 100-repeat 5-fold cross validation and hold-out performance was determined through taking the mean number of votes for each hold-out sample across all 100 repeats. Within each plot four different ROCs are represented showing the classification accuracy using ASVs or genera normalized with either total-sum-scaling or center-log-ratio abundance. Shaded areas represent 95% confidence intervals determined through 2000 bootstrap samplings.

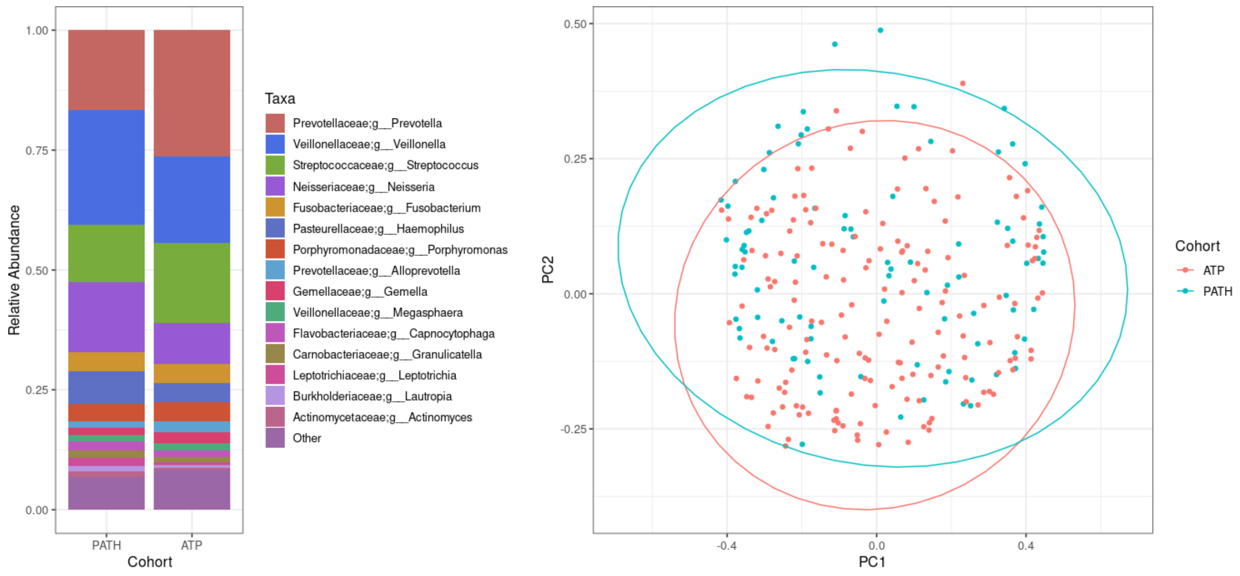

Supplemental Figure 25. Oral microbiome profiles of the non-cancer controls within the ATP and PATH cohorts. Stacked bar plot showing the mean relative abundance of genera within each cohort. All genera below 0.01 mean relative abundance were grouped into “Other”. Bray-Curtis dissimilarity profiles of overlapping genera between the ATP and PATH datasets were visualized using PCoA (PERMANOVA, p=0.001, r^2=0.03).

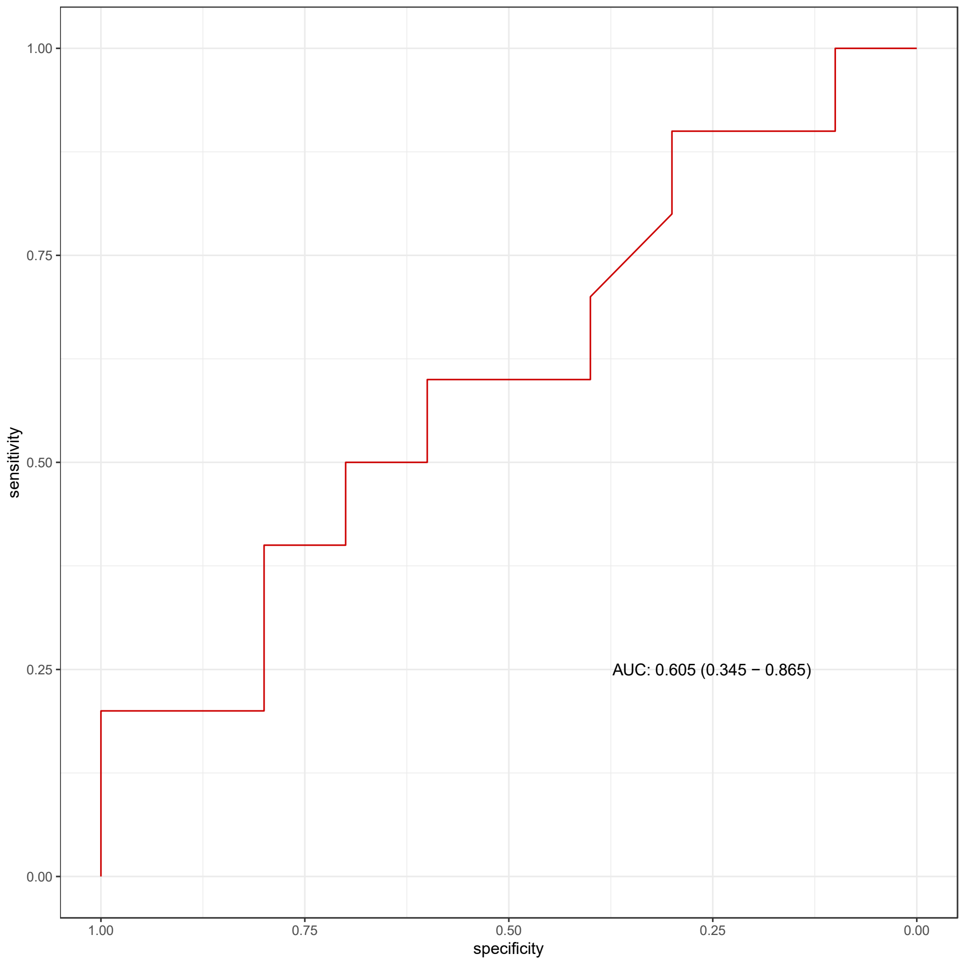

Supplemental Figure 26. Random Forest classification of prospective cases of colon cancer from a model trained on retrospective colon cancer cases. Receiver operator curves (ROC) showing the specificity and sensitivity of the classification of non-cancer matched controls and prospective cases of colon cancer within the PATH cohort. This Random Forest model was trained on the entire retrospective colon cancer dataset within the PATH cohort and then test on the entire prospective colon cancer dataset with the PATH cohort. Shaded areas represent 95% confidence intervals determined through 2000 bootstrap samplings.
